# Multi-omics Profiling Reveals the Effects of Xihuangcao on Hepatic Fibrosis Attenuation through Gut Microbiota and Metabolic Reprogramming

**DOI:** 10.34133/bmr.0422

**Published:** 2026-10-19

**Authors:** Weiguo Zhao, Xueling Zhang, Lifan Xu, Mianfeng Zheng, Zhuoya Li, Yuexuan Wang, Xinxiang He, Min Hong, Huiye Zhang, Junwei Li, Lei Shi, Ying Du, Haitao Yuan, Wei Zhang, Ali Chen

**Affiliations:** ^1^ Department of Pharmacy, Zhongshan City People’s Hospital, Zhongshan 528403, China.; ^2^ Center for Drug Research and Development, Guangdong Provincial Key Laboratory of Advanced Drug Delivery System, Guangdong Pharmaceutical University, Guangzhou 510006, China.; ^3^ Key Laboratory of Glucolipid Metabolic Disorder, Ministry of Education, Guangdong Pharmaceutical University, Guangzhou 510006, China.; ^4^ Department of Gastroenterology, The First Affiliated Hospital of Guangdong Pharmaceutical University, Guangzhou 510080, China.; ^5^ The First Affiliated Hospital of Guangdong Pharmaceutical University, Guangzhou, 510080, China.; ^6^ Hutchison Whampoa Guangzhou Baiyunshan, Chinese Medicine Company Limited, Guangzhou 510006, China.; ^7^ Consun Pharmaceutical Group Limited, Guangzhou, Guangdong 510006, China.

## Abstract

In various chronic liver diseases, hepatic fibrosis represents a frequent pathological event. This process involves the activation of hepatic stellate cells, along with the consequent excessive deposition of extracellular matrix. Emerging evidence highlights the contribution of gut microbiota dysbiosis and intestinal barrier dysfunction to its progression, underscoring the potential of targeting the liver–gut axis. Xihuangcao, a traditional Chinese medicinal herb used for acute cholecystitis and jaundice hepatitis, exhibits antifibrotic activity, but its underlying mechanisms remain unclear. In this study, hepatic fibrosis models were established to evaluate the antifibrotic effects of 3 botanical sources of Xihuangcao (*Isodon lophanthoides* (Buch.-Ham. ex D.Don) H.Hara, *Isodon lophanthoides* var. *graciliflorus* (Benth.) H.Hara, and *Isodon serra* Kudô) through integrating histopathological, transcriptomics, metabolomics, 16S ribosomal RNA (rRNA) sequencing, and network pharmacology analyses. The results indicated that all 3 botanical sources of Xihuangcao markedly alleviated hepatic fibrosis and reduced collagen deposition. Transcriptomic analysis revealed that Xihuangcao down-regulated profibrotic mediators (Toll-like receptor 5, transforming growth factor-β, and platelet-derived growth factor) and modulated mitogen-activated protein kinase, steroid biosynthesis, bile secretion, and retinol metabolism pathways, highlighting its coordinated suppression of inflammation-driven fibrogenic activation in hepatic fibrosis. Liver metabolomics revealed Xihuangcao’s effect on restoration of energy metabolism, amino acid turnover, and redox homeostasis. Xihuangcao also corrected fibrosis-induced gut dysbiosis, characterized by reduced Verrucomicrobia and increased S24-7 abundance, while suppressing pathogenic genera such as *Clostridium* and *Prevotella*. Moreover, Xihuangcao decreased profibrotic metabolites (androstan-3α,17β-diol) and suppressed choline-related metabolic pathways associated with fibrogenic signaling. Network pharmacology and experimental validation further identified PI3K/Akt inhibition as a key mechanism suppressing hepatic stellate cell activation. In summary, this study, based on integrative multi-omics analyses (metabolomics, transcriptomics, and 16S rRNA sequencing), found that Xihuangcao alleviates liver fibrosis by modulating inflammatory responses, metabolic disorders, and gut microbiota dysbiosis via the gut–liver axis. Furthermore, network pharmacology combined with in vitro experiments revealed that inhibition of hepatic stellate cell activation through the PI3K/Akt pathway may be the critical mechanism underlying its therapeutic efficacy.

## Introduction

Hepatic fibrosis (HF) constitutes a dynamic pathophysiological state marked by the aberrant buildup of extracellular matrix (ECM). This condition largely arises from persistent injuries, including viral infection, alcoholic liver disease, nonalcoholic fatty liver disease, autoimmune disorders, and genetic diseases [1]. According to recent investigations, nearly one-quarter of individuals worldwide suffer from chronic liver disease, with the global incidence of HF rising steadily [2]. Despite the liver’s intrinsic regenerative ability, which histologically allows for the reversibility of HF, timely detection and therapeutic intervention remain essential to avert disease advancement. Although strides have been made in diagnosing and managing this condition, no pharmacological agent is presently approved specifically for treating HF in a clinical setting [3].

Recent evidence has identified the gut microbiota as a pivotal modulator of hepatic physiology, contributing critically to bile acid synthesis and uptake, immune regulation within the liver, and metabolic balance [4–6]. Conversely, a growing body of research points to gut microbiota dysbiosis as an increasingly recognized factor in the initiation and exacerbation of hepatic diseases [7,8]. Moreover, HF frequently coexists with intestinal microbial imbalance and a compromised gut barrier [9]. Such microbial disruption can damage the intestinal endothelial barrier, thereby facilitating the translocation of bacterial components, such as lipopolysaccharides [10]. This translocation, in turn, may intensify hepatic inflammation and expedite the progression of HF. In light of the tight correlation between HF and the diversity of the gut microbiota, therapeutically targeting this microbial ecosystem has become an attractive approach for preventing and managing liver disorders associated with HF [11].

In recent years, traditional Chinese medicine has gained much attention for its potential in the treatment of chronic metabolic diseases, including HF. Xihuangcao, a traditional Taoist medicinal herb widely used in Lingnan, China, is documented in classical Chinese pharmacopeia for treating acute cholecystitis, acute jaundice hepatitis, and related conditions with its dried aboveground parts as medicinal parts. Despite Xihuangcao’s historical use and its reported antifibrotic effects [12], the pharmacological basis and molecular mechanisms underlying its efficacy in HF remain poorly understood. Pharmacognostic studies and ethnobotanical records identify 3 botanical sources of Xihuangcao: *Isodon lophanthoides* (Buch.-Ham. ex D.Don) H.Hara (XW), *Isodon lophanthoides* var. *graciliflorus* (Benth.) H.Hara (XH), and *Isodon serra* Kudô (XHC). The World Flora Online confirms all 3 as valid botanical names (http://www.worldfloraonline.org, accessed on 2025 June 26). However, previous studies have reported substantial variation in the chemical composition of Xihuangcao samples derived from different botanical sources, raising the question of whether their antifibrotic effects also differ, which is an issue that warrants further investigation. Although Xihuangcao exhibits antifibrotic effects, the role of the gut microbiota and its metabolism in mediating its hepatoprotective actions remains unclear.

This study aims to investigate the potential therapeutic efficacy of Xihuangcao in HF, with a specific emphasis on liver metabolism and gut microbiota. We first established a carbon tetrachloride (CCl_4_)-induced mouse model of HF, which closely mimics the pathological features observed in human liver fibrosis [13], and subsequently evaluated the hepatoprotective efficacy conferred by the 3 botanical origins of Xihuangcao through histopathological staining. Mechanistic studies underlying HF amelioration were further explored using transcriptome RNA sequencing (RNA-seq), metabolomics, and 16S ribosomal RNA (rRNA) sequencing of gut microbiota. Meanwhile, a combination of network pharmacology and experimental validation was undertaken to pinpoint the principal bioactive constituents, relevant signaling pathways, and therapeutic targets through which Xihuangcao influences liver metabolism. The results demonstrate that Xihuangcao markedly ameliorates HF. Such beneficial effects likely stem from its capacity to remodel the gut microbiota composition, reprogram metabolic routes, and suppress the expression of fibrosis-associated genes. Collectively, these findings furnish multi-omics evidence supporting the efficacy of Xihuangcao against hepatointestinal disorders and underscore its regulatory function along the gut–liver axis (Fig. 1).

**Fig. 1.**
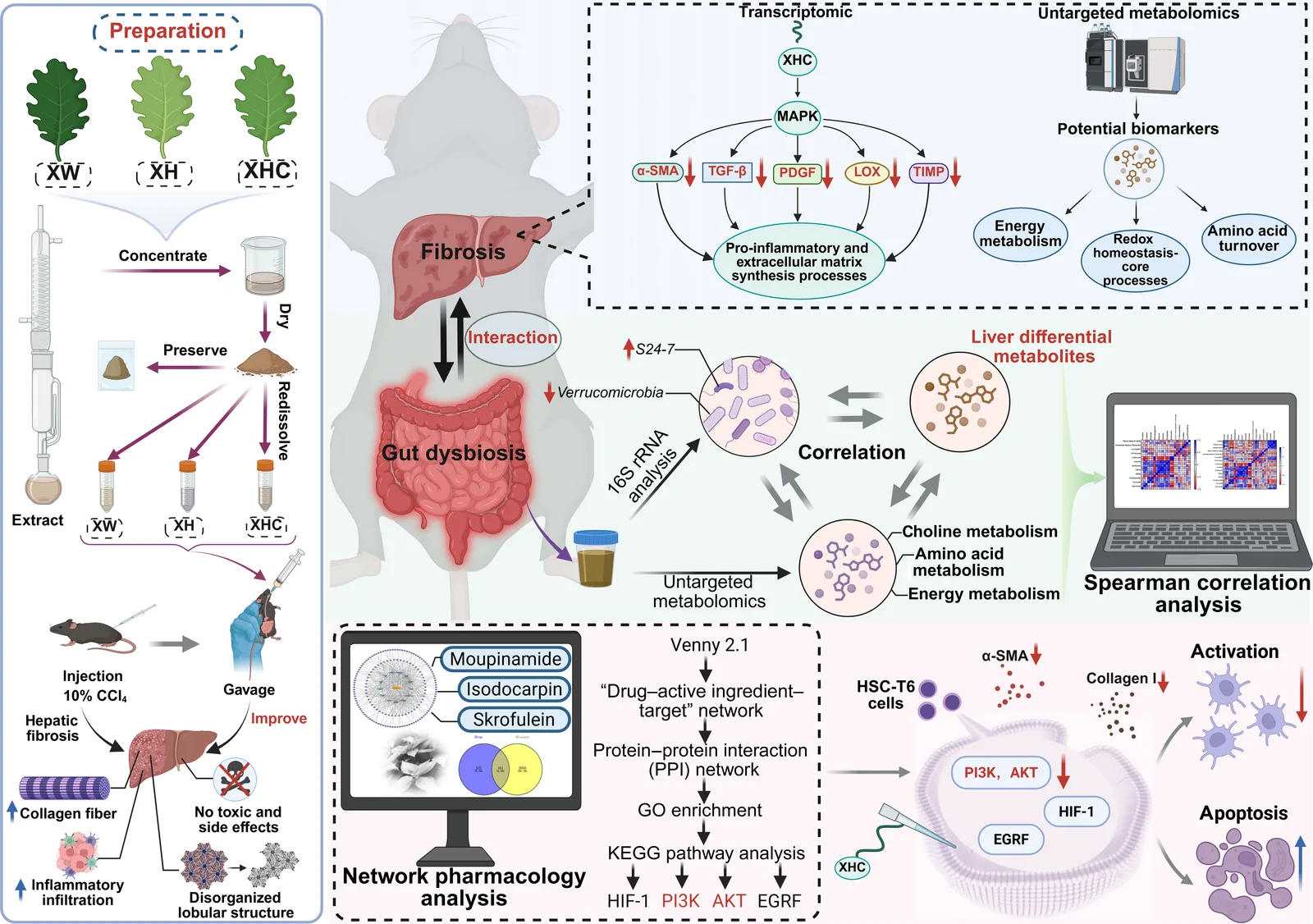
Proposed mechanisms underlying the hepatoprotection of Xihuangcao against CCl_4_-induced liver fibrosis in mice. XW, *Isodon lophanthoides* (Buch.-Ham. ex D.Don) H.Hara; XH, *Isodon lophanthoides* var. *graciliflorus* (Benth.) H.Hara; XHC, *Isodon serra* Kudô. Extracts from 3 Xihuangcao sources (XW, XH, and XHC) were administered to mice with hepatic fibrosis induced by 10% CCl_4_. Xihuangcao alleviated liver injury and fibrosis, reducing collagen, inflammatory infiltration, and structural disruption without toxicity. Multi-omics (16S ribosomal RNA [rRNA] sequencing and untargeted metabolomics) showed that Xihuangcao corrected gut dysbiosis (altered *Clostridium* and *Prevotella* abundance) and modulated hepatic metabolites linked to choline, amino acid, and energy metabolism. Spearman correlation linked gut microbiota with liver metabolites. Liver transcriptomics revealed that Xihuangcao suppressed pro-inflammatory and extracellular matrix (ECM) synthesis pathways via mitogen-activated protein kinase (MAPK), transforming growth factor-β (TGF-β), platelet-derived growth factor (PDGF), lysyl oxidase (LOX), and tissue inhibitor of metalloproteinase (TIMP) signaling. Network pharmacology identified active components (moupinamide, isodocarpin, and skrofulein) and key targets (hypoxia-inducible factor-1 [HIF-1], PI3K, Akt, and epidermal growth factor receptor [EGFR]). In HSC-T6 cells, Xihuangcao inhibited PI3K/Akt and HIF-1 signaling, reducing alpha-smooth muscle actin (α-SMA) and collagen expression and promoting apoptosis, thereby attenuating hepatic stellate cell activation and fibrogenesis.

## Materials and Methods

### Preparation of Xihuangcao extracts

Xihuangcao was procured from Guangzhou Baiyunshan Hutchison Whampoa Traditional Chinese Medicine Company Limited and verified by Senior Experimentalist Tian Suying (School of Pharmaceutical and Chemical Engineering, Guangdong Pharmaceutical University). The voucher specimen (No. XHC-2022-05) is kept at the Center for Drug Research and Development, Guangdong Pharmaceutical University. Xihuangcao extract was prepared and characterized as described in our previous study. An appropriate amount of the 3 origins of Xihuangcao powder was extracted twice with 95% ethanol (10:1, v:v) under reflux at 80 °C for 1.5 h each time and then concentrated, dried, and redissolved in pure water to obtain the 3 origins of Xihuangcao extract [14].

### Animal experiments

Male C57BL/6J mice (8 weeks old, specific-pathogen-free) were obtained from Henan Skebes Bio-technology Co. (License No.: SCXK (Yu) 2020-0005). The protocol was approved by the Guangdong Pharmaceutical University Animal Ethics Committee (No. gdpulacspf2022330). The mice were housed at the university’s Laboratory Animal Centre (License No.: SYXK (GD) 2022-0125) under controlled conditions.

After 1 week of acclimation, the mice were randomized into 5 groups (*N* = 6): control, model, XW, XH, and XHC. The control group received olive oil only; the other 4 groups were injected with 10% CCl_4_ in olive oil (1 μl/g body weight) twice weekly for 8 weeks. The control and model groups received sodium carboxymethylcellulose (200 μl/d) by gavage, while the XW, XH, and XHC groups received the corresponding herbal extracts (10.275 g/kg) for 8 weeks [15]. Additionally, this dose corresponds to the clinically equivalent dose based on body surface area conversion from traditional use.

At treatment end, the mice were anesthetized with 3% pentobarbital (intraperitoneal). Blood was collected postlaparotomy into 1.5-ml tubes, and serum was separated by centrifugation (4,500 rpm, 12 min) and stored at −80 °C. Liver tissue, feces, and cecal contents were also harvested for subsequent analyses.

### Network pharmacology analysis

The active ingredients of XHC were searched in the Traditional Chinese Medicine Systems Pharmacology (TCMSP) and HERB databases using “XHC” as the keyword. Candidate compounds and their targets were screened by oral bioavailability (OB ≥ 30%) and drug-likeness (DL ≥ 0.18). HF-related targets were retrieved from DisGeNET and GeneCards with the term “HF”. After merging and deduplication, the intersecting drug–disease targets were obtained. These targets, along with drug and ingredient data, were imported into Cytoscape 3.9.1 to build an “XHC–ingredient–target” network. The main active components against HF were prioritized by degree value. Gene Ontology (GO) analysis (covering Biological Process, Cellular Component, and Molecular Function) and Kyoto Encyclopedia of Genes and Genomes (KEGG) pathway analysis were performed via the DAVID database using the intersecting targets and species setting of *Homo sapiens*. Terms with *P* < 0.05 were sorted by *P* value (ascending), and the top 20 were visualized as bubble charts using WeChat online tools.

### NMR spectral experiments

Liver ^1^H nuclear magnetic resonance (^1^H-NMR) spectra were acquired on a Bruker AVANCE III 500-MHz spectrometer at 298 K using a Carr–Purcell–Meiboom–Gill pulse sequence (total echo time 100 ms, relaxation delay 3 s, spectral width 10 kHz, 128 scans). Data were processed with MestReNova. Chemical shifts were referenced to the lactate doublet at *δ* 1.33 ppm. Automatic integration was performed from 0.8 to 9.0 ppm (0.01-ppm intervals). Methanol (3.34 to 3.38 ppm) and water (4.70 to 5.10 ppm) signals were removed, and the remaining spectra were normalized to a total area of 1.

Multivariate analysis of normalized data was conducted using SIMCA-P 14.0. Principal component analysis (PCA) was used to assess sample clustering and raw data classification. Model validity was tested by 200 permutation tests. Metabolite differences between groups were evaluated using orthogonal partial least squares discriminant analysis (OPLS-DA) variable importance in projection (VIP) values. Pathway analysis was performed via MetaboAnalyst 5.0 by matching 21 differential metabolites against the Human Metabolome Database for enrichment and topology analysis.

### Analysis conditions of fecal liquid chromatography–quadrupole time-of-flight MS

Chromatography was performed on a Waters Acquity I-Class PLUS UPLC system with an HSS T3 column (1.8 μm, 2.1 × 100 mm). Mobile phases: A = 0.1% formic acid in water; B = 0.1% formic acid in acetonitrile. Injection: 2 μl; flow rate: 400 μl/min. Gradient: 0 to 0.5 min (95% A), 0.5 to 5.5 min (95% to 50% A), 5.5 to 9.0 min (50% to 5% A), 9.0 to 10.5 min (5% A), and 10.5 to 12.0 min (5% to 95% A) [16]. Mass spectrometry (MS) analysis used Waters Xevo G2-XS QTof controlled by MassLynx V4.2. MSe mode provided simultaneous MS^1^ and MS^2^ data. Electrospray ionization parameters: capillary voltage ±2,500/−2,000 V, cone 30 V; source temperature 100 °C, desolvation 500 °C; desolvation gas 800 l/h, cone gas 50 l/h; *m*/*z* range 50 to 1,200. Data were processed with Progenesis QI (peak detection and retention time alignment). Compound identification relied on the METLIN database, public libraries, an in-house spectral library, and theoretical fragment prediction.

### 16S rRNA gene sequencing

Feces collected at weeks 3, 6, and 8 were stored at −80 °C. Gut microbial genomic DNA was extracted according to a published method [17]. In brief, fecal samples were dissolved in phosphate-buffered saline with 0.5% Tween 20 and then freeze–thawed (−80 °C for 10 min and 60 °C for 5 min) 3 times. DNA was purified by phenol–chloroform–isoamyl alcohol extraction. The V3–V4 region of the 16S rRNA gene was amplified with primers V4-F (5′-GTGTGYCAGCMGCCGCGGTAA-3′) and V4-R (5′-CCGGACTACNVGGGTWTCTAAT-3′). Sequencing was conducted on Illumina HiSeq PE250, and raw data were processed with QIIME 2 (v2020.2).

### Western blot

Total liver proteins were isolated in radioimmunoprecipitation assay buffer, resolved by 10% sodium dodecyl sulfate–polyacrylamide gel electrophoresis, and blotted onto polyvinylidene fluoride membranes. After blocking for 1 h at room temperature in 5% nonfat milk/tris-buffered saline with Tween 20 (0.1% Tween 20), the membranes were probed with primary antibodies at 4 °C overnight, followed by a 1-h incubation with horseradish peroxidase-linked secondary antibodies (1:10,000). Protein bands were quantified by densitometry using ImageJ (v1.52), and all target protein signals were normalized against glyceraldehyde-3-phosphate dehydrogenase to control for loading variations (Table S1).

### Statistical analysis

Data were analyzed with GraphPad Prism 8. An unpaired 2-tailed *t* test or one-way analysis of variance with Bonferroni’s correction was used for comparisons. Results are reported as mean ± standard error of the mean, with *P* < 0.05 considered significant. *P < 0.05, **P < 0.01, ***P < 0.001, and ****P < 0.0001.

## Results

### The 3 botanical sources of Xihuangcao exhibit differential efficacy against CCl_4_-induced HF in mice

In this study, male 8-week-old C57BL/6J mice were used to explore the protective effects of extracts from the 3 origins of Xihuangcao against CCl_4_-induced HF. The experimental design and timeline are illustrated in Fig. 2A. Compared with those in the control mice, the serum levels of aspartate aminotransferase (AST) and alanine aminotransferase (ALT) were significantly elevated in the model group, whereas Xihuangcao treatment remarkably reduced these abnormal aminotransferase levels. Notably, the serum levels of AST were significantly reduced only in the XW group, whereas ALT levels were significantly decreased in both the XH and XHC groups (Fig. 2B and C). Histological analysis of liver tissue sections was conducted using hematoxylin and eosin staining, Masson trichrome staining, Sirius red staining, and immunohistochemical (IHC) staining to assess the extent of HF. In the control group, the hepatocytes were orderly arranged, the structure of the hepatic lobules were intact, central veins were radially distributed, and no collagen fibers were observed. In contrast, the model group exhibited a disorganized lobular structure, abundant collagen deposition surrounding hepatocyte clusters, prominent inflammatory cell infiltration, and increased blue-stained (collagen) and red-stained (type I collagen) fibers in Masson and Sirius red staining. Treatment with Xihuangcao (XW, XH, and XHC groups) all alleviated these pathological changes by reducing inflammatory infiltration, restoring lobular architecture, and decreasing collagen fiber deposition. Among the treatment groups, the XH group exhibited the least collagen accumulation based on Masson and Sirius red staining (Fig. 2D to H). To further evaluate the degree of HF and assess the activation status of hepatic stellate cells (HSCs), IHC staining for alpha-smooth muscle actin (α-SMA), a well-established marker of stellate cell activation, was performed. In the IHC analysis, cell nuclei were stained blue, while α-SMA-positive signals appeared as brown areas. No α-SMA staining was observed in the control group, whereas the model group exhibited extensive brown coloration, indicating pronounced activation of HSCs. Following drug administration, the brown-staining areas were markedly reduced, with the XW-treated group showing the lowest α-SMA positivity (Fig. 2I and J), suggesting a potent inhibitory effect on HSC activation. To evaluate the potential toxicity of Xihuangcao treatment, a toxicological experiment was performed and showed that the administration of Xihuangcao extract alone (without modeling) had no significant effect on the liver function and other physiological indicators of the experimental animals, indicating that Xihuangcao extract has good safety (Fig. S1A to F). Compared with colchicine in the positive control group, Xihuangcao extract improved HF. The above results indicate that all 3 extracts derived from Xihuangcao exhibited varying degrees of hepatoprotective activity and effectively suppressed CCl_4_-induced HF in mice. Moreover, the safety evaluation demonstrated that *Rabdosia serra* extracts possess favorable safety profiles, providing important toxicological evidence to support long-term oral administration and potential clinical application.

**Fig. 2.**
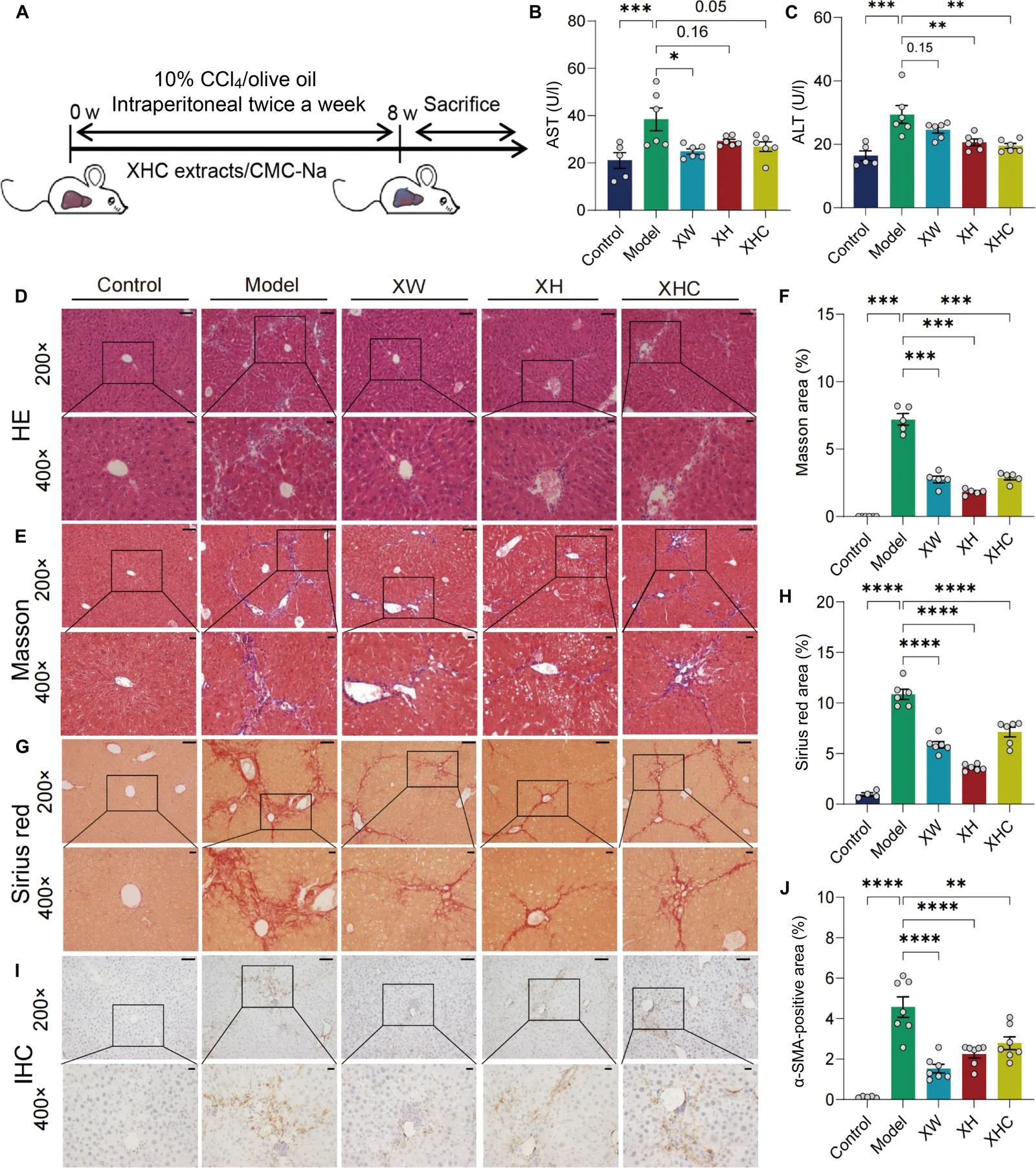
The 3 botanical sources of Xihuangcao exhibit differential efficacy against CCl_4_-induced hepatic fibrosis in mice. The abbreviations XW, XH, and XHC are defined in Fig. 1. (A) Flowchart of the experimental protocol of Xihuangcao. (B and C) Serum alanine aminotransferase (ALT) and aspartate aminotransferase (AST) levels in mice (*N* = 5 to 6). (D) Hematoxylin and eosin (HE) staining diagram of liver tissue (*N* = 5 to 7). (E and F) Masson staining graph of liver tissue and analysis of results (*N* = 5 to 6). (G and H) Sirius red staining diagram of liver tissue (*N* = 4 to 7). (I and J) Immunohistochemical staining and quantitative analysis of alpha-smooth muscle actin (α-SMA) expression in liver tissues (*N* = 5 to 7). All data are shown as mean ± standard error of the mean (SEM). Magnification 200×, scale bar 100 µm; magnification 400×, scale bar 40 µm.

### Xihuangcao reshapes the liver transcriptome and modulates inflammatory pathways

To gain deeper mechanistic insights into the hepatoprotective action of XHC, we performed transcriptome-wide RNA-seq on liver tissues. Unsupervised PCA revealed a robust segregation of the control, model, and XHC groups along the first 2 principal components (PC1 and PC2, Fig. 3A), indicating that XHC intervention induced a substantial reprogramming of the hepatic transcriptional network in the context of CCl_4_-driven fibrogenesis. The clustering heat map further showed distinct gene expression patterns between the control, model, and XH groups, with the XHC group remodeling the expression trends of several fibrosis-associated genes in the model group, suggesting its regulatory effect at the transcriptional level (Fig. 3B). The volcano plot revealed distinct patterns of gene expression alterations across groups. Compared with the control group, the model group exhibited 1,677 significantly up-regulated and 1,180 down-regulated genes, demonstrating that carbon tetrachloride treatment markedly perturbed the hepatic transcriptome. In contrast, the XHC group showed 366 up-regulated and 1,267 down-regulated genes relative to the model group, indicating its broad modulatory effects across multiple pathways (Fig. 3C and D). Pathway enrichment analysis showed that XHC significantly regulates multiple pathways related to liver fibrosis, such as the steroid biosynthesis pathway, the bile secretion pathway, and negative regulation of the mitogen-activated protein kinase (MAPK) cascade pathway (Fig. 3E), indicating that XHC may exert an anti-HF effect by regulating these pathways. The down-regulated genes in the differentially expressed gene heat map include Toll-like receptor 5 (TLR5), Tef, and Per3, and the up-regulated genes include Slc45a3 and Arntl (Fig. 3F). Among them, TLR5 is directly related to the activation of the MAPK pathway. Therefore, XHC may exert an anti-liver fibrosis effect by specifically inhibiting the excessive activation of the negative regulation of the MAPK cascade pathway, thereby down-regulating the expression of profibrotic factors (transforming growth factor-β [TGF-β] and platelet-derived growth factor [PDGF]) and reducing the deposition of collagen.

**Fig. 3.**
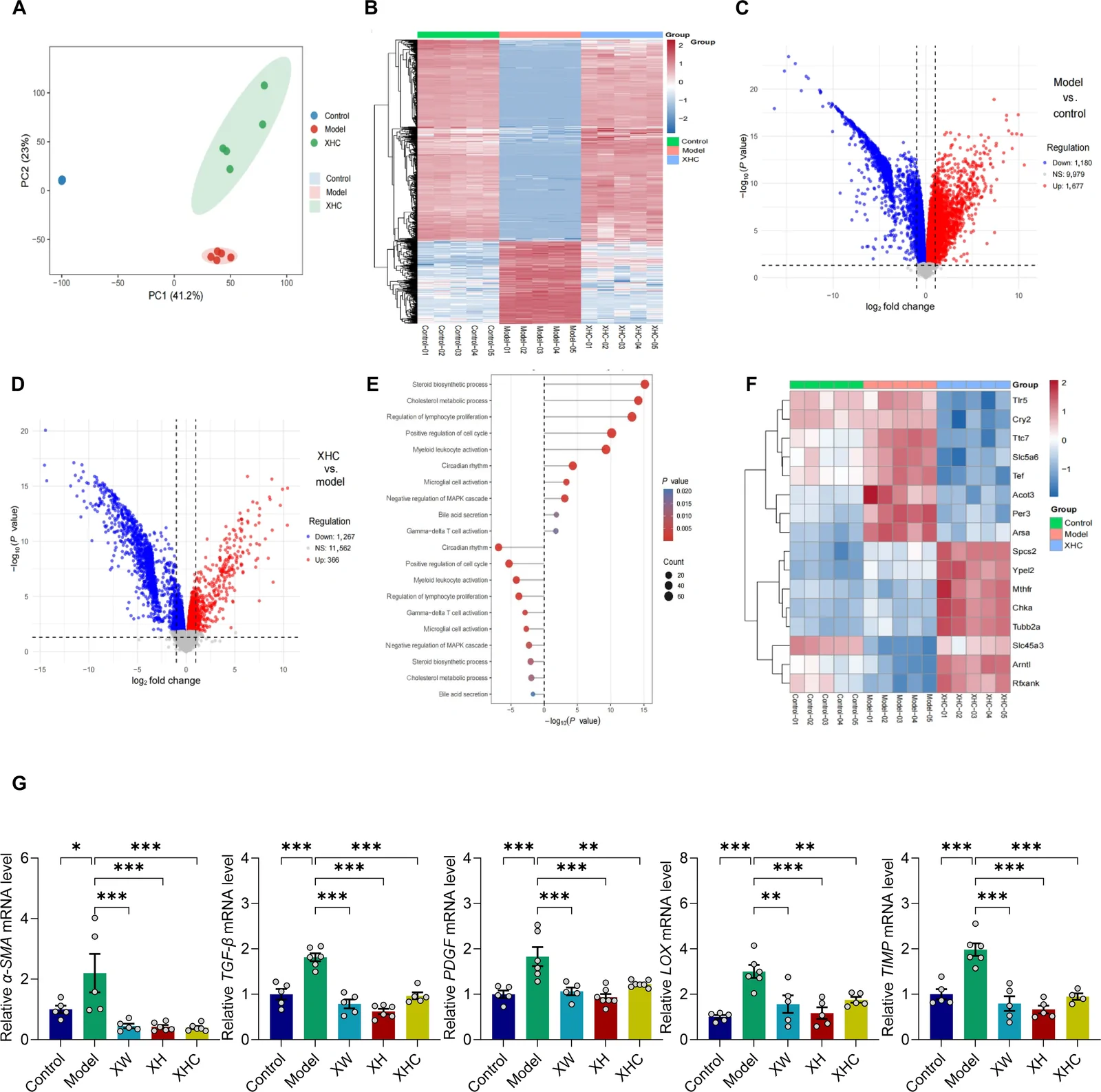
Xihuangcao restores the liver transcriptomic profile in mice with hepatic fibrosis. (A) Principal component analysis (PCA) score plot. (B) Differentially expressed gene heat map. (C) Differential gene volcano map between the control and model groups. (D) Differential gene volcano map between the model and *Isodon serra* Kudô (XHC) groups. (E) Bubble chart of pathway enrichment analysis. (F) Heat map of differentially expressed genes. (G) Hepatic tissue *α-SMA*, *TGF-β1*, *PDGF*, *LOX*, and *TIMP* messenger RNA (mRNA) gene expression (*N* = 4 to 7). All data are shown as mean ± standard error of the mean (SEM).

Based on the findings from pathological studies and transcriptomic analyses, we performed quantitative reverse transcription polymerase chain reaction (RT-PCR) on liver tissues to evaluate the effects of Xihuangcao from the 3 different sources. Quantitative RT-PCR analysis of liver tissues further confirmed that fibrotic markers (*α-SMA*, *TGF-β*, *PDGF*, *LOX*, and *TIMP*) were significantly elevated in the model group but markedly reduced after Xihuangcao treatment, suggesting that its antifibrotic effects involve inhibition of fibrotic signaling and enhanced matrix degradation (Fig. 3G). Collectively, these transcriptomic findings indicate that XHC exerts antifibrotic effects by reshaping gene expression profile, regulating key fibrotic markers, and intervening in key metabolic and inflammatory signaling pathways.

### Xihuangcao restores the liver metabolites of mice with HF

To investigate the effects of Xihuangcao treatment on hepatic metabolism, an untargeted metabolomics analysis was performed on mouse liver tissues. Representative ^1^H-NMR spectra and raw data of liver samples from the control, model, and XW, XH, and XHC groups were processed using the MestReNova software (Fig. S2). To identify metabolic alterations induced by the 3 origins of Xihuangcao, we performed PCA and OPLS-DA. Clear separation between the control and model groups in both PCA and OPLS-DA score plots (Fig. 4A and B), indicated distinct liver metabolic profiles due to CCl_4_-induced fibrosis. The reliability of the OPLS-DA models was further confirmed by permutation testing (Fig. 4C).

**Fig. 4.**
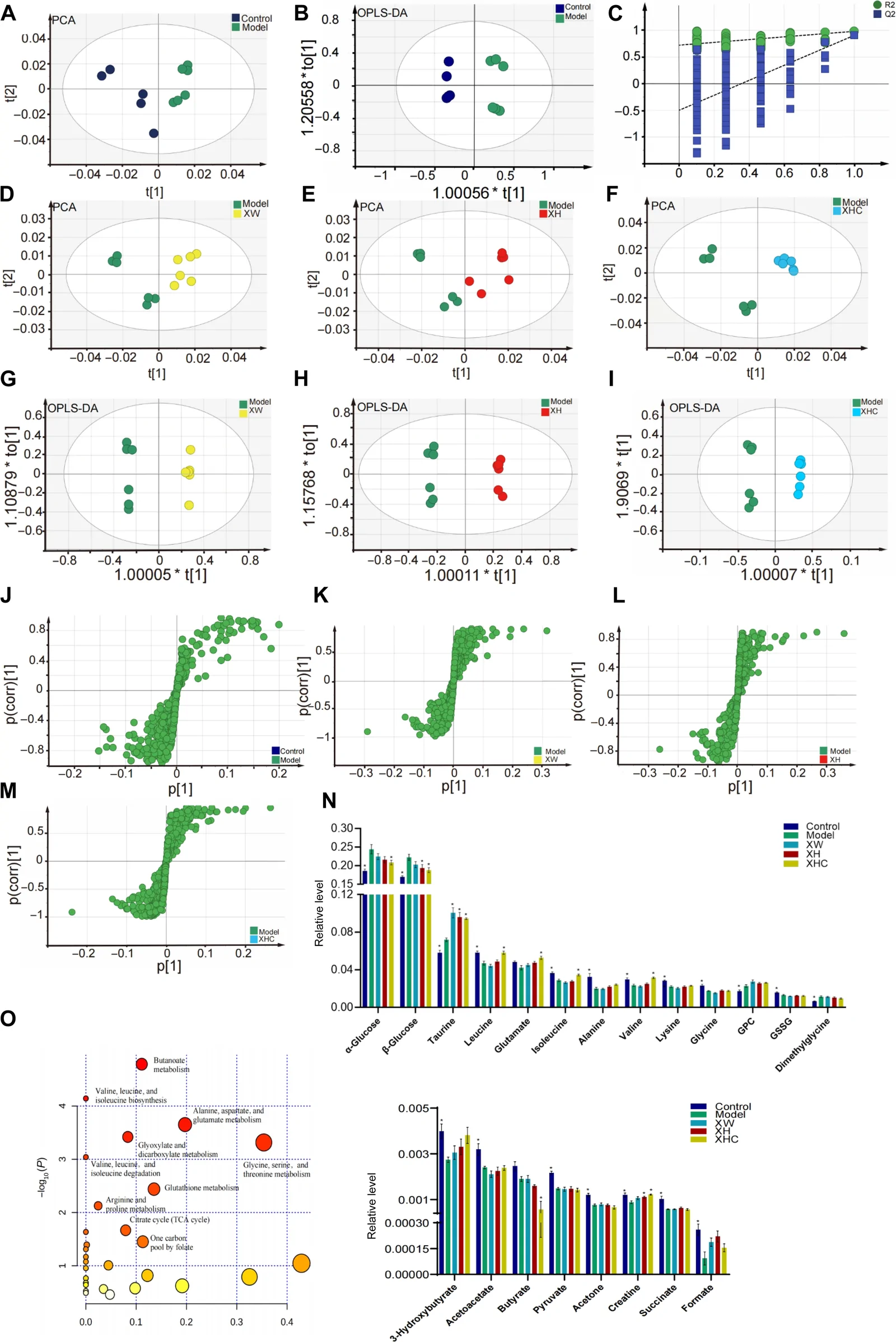
Xihuangcao restores liver metabolites of mice with hepatic fibrosis and multivariate analysis of liver metabolomics. The abbreviations XW, XH, and XHC are defined in Fig. 1 (A) Principal component analysis (PCA) plot of the control group vs. the model group. (B) Orthogonal partial least squares discriminant analysis (OPLS-DA) plot of the control group vs. the model group. (C) 200 permutation tests of the control and model groups; 200 random permutations confirmed that no overfitting occurred in the model. (D) PCA plot of the model group vs. the XW group. (E) PCA plot of the model group vs. the XH group. (F) PCA plot of the model group and the XHC group. (G) OPLS-DA score plot of the model group vs. the XW group. (H) OPLS-DA score plot of the model group vs. the XH group. (I) OPLS-DA score plot of the model group vs. the XHC group. (J) S-plots for the control group vs. the model group. (K to M) S-plots for the model group vs. the administration group. (N) Relative levels of differential metabolites in mouse liver tissue (*N* = 5 to 6). GPC, l-α-glycerylphosphorylcholine; GSSG, oxidized glutathione. (O) Metabolic pathway analysis using MetbraAnalyst. All data are shown as mean ± standard error of the mean (SEM).

In addition, PCA and OPLS-DA score plots showed significant separation between the model group and each of the XW, XH, and XHC treatment groups along the t[1] axis, indicating that all 3 origins of Xihuangcao partially remodeled CCl_4_-induced HF-associated metabolic changes (Fig. 4D to I). The S-plots derived from the OPLS-DA model (Fig. 4J to M), along with loading plots and univariate statistical analysis (Student *t* tests), were used to screen potential biomarkers.

Twenty-one significantly altered endogenous metabolites in liver tissues were identified as potential biomarkers based on VIP values (VIP ≥ 1), including α-glucose, β-glucose, taurine, leucine, glutamate, isoleucine, alanine, valine, lysine, glycine, l-α-glycerylphosphorylcholine, oxidized glutathione, dimethylglycine, 3-hydroxybutyrate, acetoacetate, butyrate, pyruvate, acetone, creatine, succinate, and formate (Fig. 4N and Table S2). Nine metabolic pathways with impact values >0.1 were identified as significantly altered in the HF model and partially restored after Xihuangcao administration (Fig. 4O and Table S3). These pathways are associated with energy metabolism, amino acid turnover, and redox homeostasis-core processes disrupted in liver fibrosis.

### Xihuangcao modulates the gut microbiota of mice with HF

Increasing evidence indicates that abnormal alterations in gut microbiota have been closely associated with the onset and progression of liver fibrosis [4]. To gain deeper insights into the mechanism by which Xihuangcao alleviates liver fibrosis, we conducted 16S rRNA sequencing analysis of gut microbiota using the fecal samples of mice. We assessed both alpha diversity (α-diversity) and beta diversity (β-diversity) in fecal samples after 8 weeks of administration. Alpha diversity reflects the species richness and evenness within a microbial community. In this study, the Shannon index, Faith’s phylogenetic diversity (PD) index, and observed features index were employed for analysis. No significant differences in any of the 3 indices were observed between the Xihuangcao-treated group and the control group (Fig. 5A to C), indicating that after Xihuangcao intervention in HF, the overall richness and evenness of the gut microbiota were restored to levels comparable to those of the control group. Specifically, the Shannon index showed no significant difference in the XH group compared to the control, while the PD index and observed index were elevated, indicating a potential increase in PD. In the XW group, although PD and observed indices were similar to those of the control, the Shannon index was higher, implying increased diversity in species evenness. Beta diversity analysis based on Bray–Curtis, euclidean, unweighted-unifrac, and weighted-unifrac revealed distinct clustering among the groups evidenced by principal coordinates analysis results (Fig. 5D to G), suggesting that the microbial community structures differed significantly among the control, model, and Xihuangcao-treated groups. This suggests that Xihuangcao, including its 3 regional variants (XW, XH, and XHC), modulated the gut microbial composition to varying extents.

**Fig. 5.**
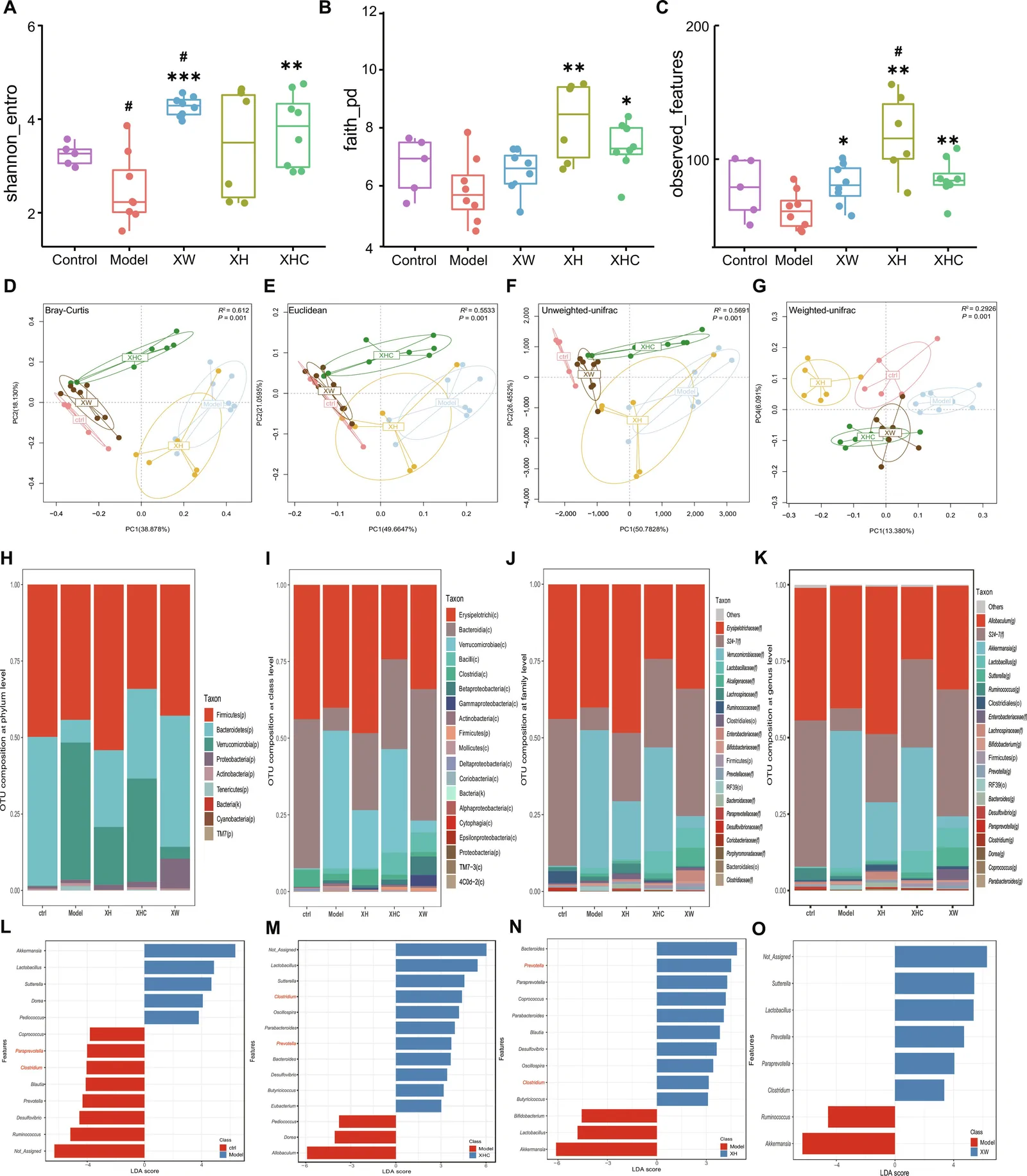
Xihuangcao modulates abnormal gut fungal microbiota of mice with hepatic fibrosis. The abbreviations XW, XH, and XHC are defined in Fig. 1. (A to C) Alpha diversity Shannon index, faith_pd index, and observed_features index at 8 weeks of administration (*N* = 5 to 8). (D to G) Bray–Curtis, euclidean, unweighted-unifrac, and weighted-unifrac principal coordinates analysis (PCoA) of beta diversity. (H to K) Differential flora at the operational taxonomic unit (OTU) taxonomic phylum, class, family, and genus levels in each group. (L to O) Linear discriminant analysis (LDA) score plots of the control and model groups, model and XHC groups, model and XH groups, and model and XW groups after 8 weeks of administration. All data are shown as mean ± standard error of the mean (SEM).

Taxonomic classification from phylum to genus levels showed differences in gut microbial profiles (Fig. 5H to K). At the phylum level, Firmicutes and Bacteroidetes dominated the control group. The model group exhibited a notable decrease in Bacteroidetes, a hallmark of microbial imbalance, which would lead to reduced short-chain fatty acid production, potentially exacerbating inflammation and worsening liver injury. Expectedly, all administered groups of Xihuangcao increased Bacteroidetes levels, suggesting a restorative effect on microbial balance. There was a similar trend of the abundance of Bacteroidia at the class level (Fig. 5I). Consistently, the liver fibrosis model led to a significant reduction in the abundance of S24-7 (*Muribaculaceae*) at both the family and genus levels, indicating microbial dysbiosis associated with hepatic injury. Notably, Xihuangcao treatment restored the abundance of S24-7, suggesting a therapeutic modulation of the gut microbiota. Meanwhile, at the phylum, class, and family levels, the liver fibrosis model was associated with a significant increase in Verrucomicrobia (Fig. 5H to J), potentially reflecting mucosal barrier impairment and gut microbial dysbiosis. Xihuangcao treatment significantly reduced this increase, suggesting that it may restore gut microbial homeostasis and improve intestinal barrier function, thereby contributing to its protective effects against liver fibrosis.

To further identify differentially abundant *Enterobacteriaceae* taxa (from phylum to genus) between the groups, the linear discriminant analysis effect size method (linear discriminant analysis scores > 2.0) was performed, which revealed distinct bacterial signatures associated with each group (Fig. 5L to O). In particular, *Clostridium* and *Prevotella* were enriched in the model group and decreased following Xihuangcao treatment, indicating that Xihuangcao suppresses potentially pathogenic bacteria associated with HF.

### Multi-omics analysis reveals that XHC restores fecal metabolites in mice with HF

Although all 3 botanical sources exhibited antifibrotic activity, XHC—as the pharmacopoeial source documented in the Chinese Pharmacopoeia and the one displaying a more diverse chemical repertoire in preliminary phytochemical screening—was therefore particularly suitable for metabolomic pathway enrichment-based discovery. To evaluate the effects of XHC on intestinal metabolism in HF, fecal metabolites were profiled using liquid chromatography–quadrupole time-of-flight MS nontargeted metabolomics under both positive- and negative-ion modes. The total ion current spectra of the control group, model group, and XHC groups demonstrated consistent peak intensity and retention times, indicating the stability and reliability of the experimental system (Fig. S3).

PCA revealed distinct clustering among the 3 groups in both ion modes, indicating statistically significant differences in the composition of fecal metabolites (Fig. 6A). Subsequently, OPLS-DA was conducted to further assess data quality. Clear separation was observed between the control and model group, as well as the model group and the XHC group in the positive-ion mode and the negative-ion mode, indicating that HF alters fecal metabolites and that XHC intervention reshapes gut metabolites. The OPLS-DA model showed that the *R*^2^*Y* values are all higher than 0.9, and the *Q*^2^ values are all above 0.5, indicating that this model not only has excellent explanatory efficiency but also shows reliable predictive ability. Furthermore, the results of 200 displacement tests show that, whether in the positive-ion or negative-ion mode, the slopes of the *R*^2^*Y* and *Q*^2^ lines in the control group and the model group, as well as the model group and the XHC group, are all positive, once again proving that the model has better explanatory and predictive capabilities (Fig. 6B).

**Fig. 6.**
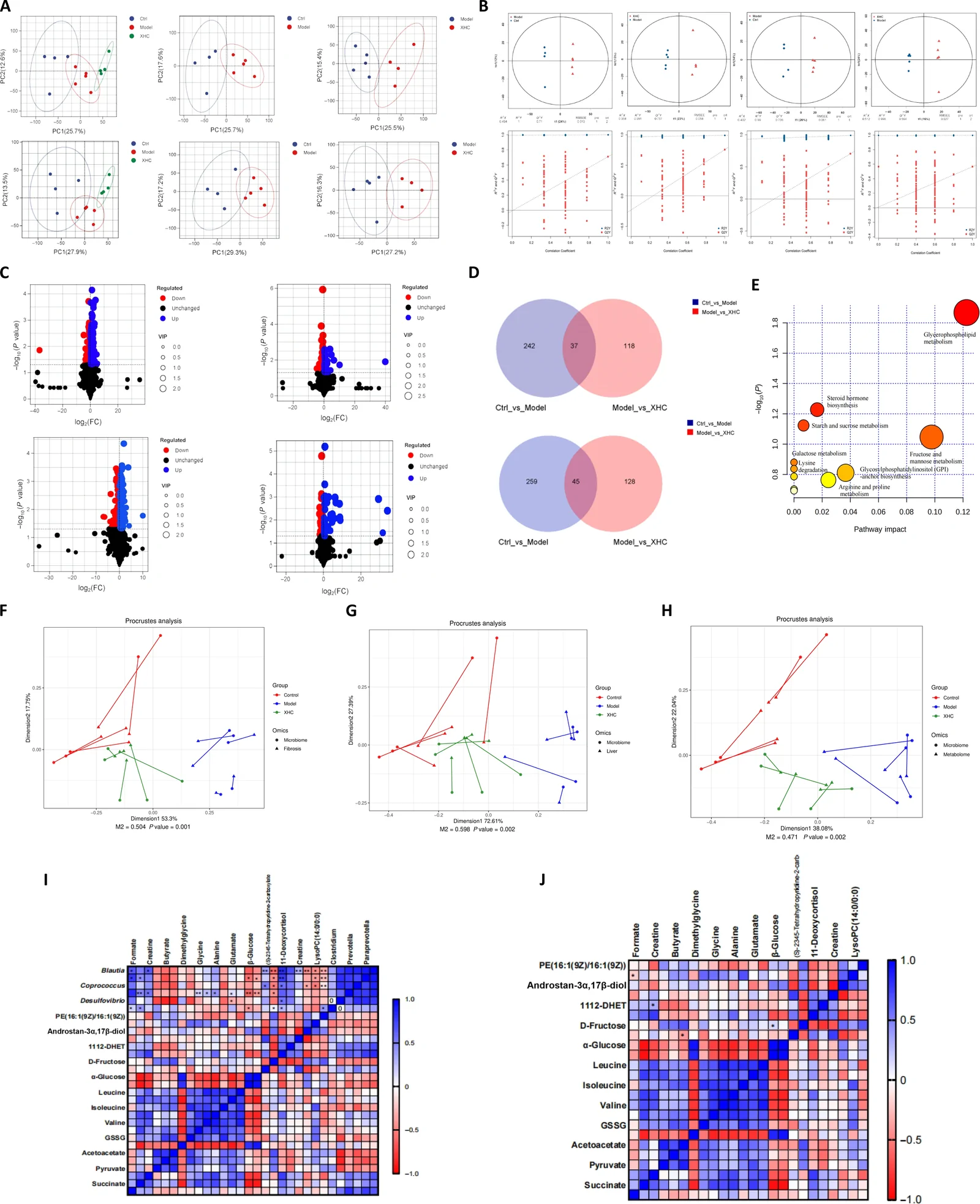
Xihuangcao induces systemic metabolic restoration in mice with hepatic fibrosis: integrated metabolomic profiling of liver and feces. (A) Principal component analysis (PCA) score plot of fecal metabolites (*N* = 4 to 6). (B) orthogonal partial least squares discriminant analysis (OPLS-DA) score plot of fecal metabolites (*N* = 4 to 6). (C) Volcano plot of fecal metabolites. (D) Venny diagram of differential metabolites in fecal metabolites. (E) Metabolic pathway analysis using MetbraAnalyst. (F) Procrustes analysis of the association between gut microbiota and fibrosis-related pathway genes. (G) Procrustes analysis of the association between gut microbiota and hepatic metabolites. (H) Procrustes analysis of the association between gut microbiota and liver function. (I) Spearman correlation analysis of gut microbiota and differential metabolites. (J) Spearman correlation analysis among differential metabolites.

Based on the OPLS-DA results, the screening of differential metabolites was conducted using VIP value > 1, *P* value ≤ 0.05, and fold change ≥ 1. A total of 2,440 and 1,892 metabolites were successfully annotated in the positive- and negative-ion modes, respectively. Volcano plots illustrated the distribution of differential metabolites by fold change and statistical significance. Metabolites with up-regulation, down-regulation, or no significant change were marked in blue, red, and black, respectively, with point size reflecting VIP values (Fig. 6C). Combining the results from control vs. model and model vs. XHC comparisons, 45 differential metabolites were screened in the positive-ion mode, and 37 in the negative-ion mode (Fig. 6D and Tables S4 and S5). In the model group, the levels of metabolites such as cholestane triol and lysophosphatidylcholine were significantly increased, while those of phenylpropionitrile and 4-*n*-butylphenol were significantly decreased. Similar trends were observed under negative-ion mode, with elevated levels of methylphosphonate ester, quine thiocyanate, etc., and reduced levels of succinamide, creatine, etc. Pathway enrichment analysis showed that these differential metabolites were mainly involved in pathways involving amino acid metabolism, choline metabolism, and energy metabolism (Fig. 6E and Table S6). Notably, the administration of XHC reshaped gut metabolites, suggesting that its antifibrotic effects may be linked to modulation of gut microbial metabolism—particularly through the inhibition of choline-related pathways.

Procrustes analysis was employed to assess the associations among gut microbiota, hepatic metabolites, and fibrosis-related pathway genes in the liver (*α-SMA*, *TGF-β*, *PDGF*, *LOX*, and *TLR5*). Significant correlations were observed between gut microbiota and fibrosis-related pathway genes (*P* < 0.01; Fig. 6F), as well as between gut microbiota and hepatic metabolites (*P* < 0.01; Fig. 6G). Additionally, a significant correlation was identified between gut microbiota and the liver (*P* < 0.01; Fig. 6H).

Spearman correlation analysis between 16S RNA-seq and metabolomics data screened by fecal and liver metabolomics revealed close connection between the gut microbial shifts and key hepatic/fecal differential metabolites. The Spearman correlation heat map can visually demonstrate the close association between the gut microbiota and key differential metabolites. The closer the absolute value is to 1, the higher the correlation. *Blautia* is positively correlated with 11-deoxycortisol, phosphatidylethanolamine (16:1(9Z)/16:1(9Z)), and creatine but negatively correlated with d-fructose, LysoPC (14:0/0:0), and androstan-3α,17β-diol. *Prevotella* is positively correlated with d-fructose, α-glucose, β-glucose, and dimethylglycine. *Prevotella* is negatively correlated with glutamate, alanine, valine, glycine, creatine, and succinate (Fig. 6I and Table S7). Meanwhile, Spearman correlation analysis was conducted on fecal differential metabolites and liver differential metabolites. It could be seen that the correlation between the differential metabolites was not high, and only a few groups of metabolites were correlated, such as leucine and valine being positively correlated, and α-glucose and butyrate being negatively correlated (Fig. 6J and Table S8).

Collectively, comprehensive multi-omics analysis reveals that *Blautia* and *Prevotella* correlated positively with branched-chain amino acids (BCAAs), suggesting their involvement in BCAA biosynthesis or conservation. Conversely, profibrotic metabolites such as androstan-3α,17β-diol were down-regulated following XHC treatment, possibly due to remodeling of the structure of the microbiota. This highlights a synergistic “microbiota–metabolite–liver axis” as a central therapeutic route through which XHC exerts its protective effects against HF.

### Network pharmacology analysis of XHC in the treatment of HF

To identify the key components, targets, and potential pathways through which XHC modulates HF, we employed a traditional Chinese medicine network pharmacology approach combined with in vitro validation. Specifically, a total of 13 active compounds in XHC and 383 related targets were identified from the TCMSP and HERB databases. A total of 2,217 disease targets were retrieved from the DisGeNET and GeneCards databases, and 161 overlapping genes associated with liver fibrosis were obtained using Venny 2.1 (Fig. 7A). Based on the 161 overlapping targets of XHC and liver fibrosis mentioned above, the relationship between XHC monomers and therapeutic targets of liver fibrosis was revealed. To visualize this relationship, Cytoscape was imported to construct a “drug–active ingredient–target” network, highlighting isodocarpin, moupinamide, and skrofulein as core active ingredients of Xihuangcao in anti-liver fibrosis (Fig. 7B). The protein–protein interaction network is then imported into Cytoscape for topological analysis, which revealed key hub targets including signal transducer and activator of transcription 3 (STAT3), heat shock protein 90α family class A member 1 (HSP90AA1), protein kinase B (AKT1), nonreceptor tyrosine kinase (SRC), tumor necrosis factor (TNF), epidermal growth factor receptor (EGFR), and B-cell lymphoblastoma-2 (BCL) 2, Janus kinase 2 (JAK2), estrogen receptor 1 (ESR1), and the catalytic subunit α of phosphatidylinositol 4,5-diphosphate 3-kinase (PIK3CA) (Fig. 7C), suggesting their central roles in XHC’s antifibrotic activity. GO enrichment through the DAVID database identified 975 terms related to the positive regulation of the MAPK cascade, the collagen catabolic process, phosphatidylinositol 3 kinase/protein kinase B (PI3K) signal transduction, negative regulation of the apoptotic process, etc. (Fig. 7D and E). KEGG pathway analysis of the first 20 pathways showed significant enrichment in EGFR tyrosine kinase inhibitor resistance, PI3K/Akt signaling pathway, hypoxia-inducible factor-1 (HIF-1) signaling pathway, etc. (Fig. 7F and G).

**Fig. 7.**
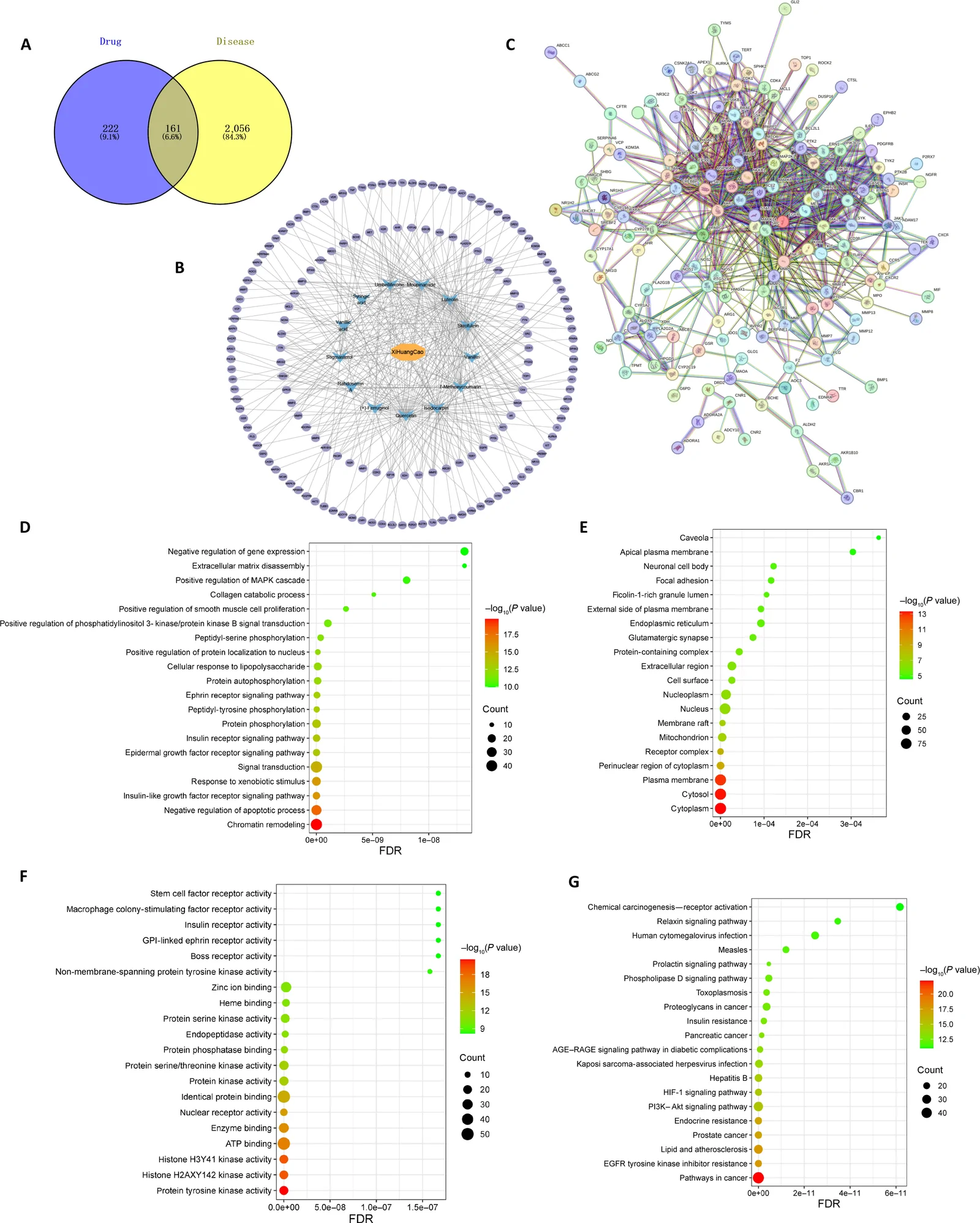
Network pharmacology analysis of *Isodon serra* Kudô (XHC) in the treatment of hepatic fibrosis. (A) Venny diagram of the intersection target of XHC and liver fibrosis. (B) “Drug–active component–target” network diagram. (C) Protein–protein interaction (PPI) network diagram. (D) Gene Ontology (GO) enrichment analysis—Biological Process (BP), Cellular Component (CC), Molecular Function (MF). (E) Kyoto Encyclopedia of Genes and Genomes (KEGG) pathway enrichment analysis of the top 20 pathways, with PI3K/Akt, epidermal growth factor receptor (EGFR) resistance, and hypoxia-inducible factor-1 (HIF-1) signaling prominently enriched. (F) Bubble charts for GO terms. (G) Bubble charts for KEGG pathways. All analyses were performed using the DAVID database (*P* < 0.05).

### XHC mitigates HF by inhibiting the PI3K/Akt signaling pathway

Based on the network pharmacology findings, we further verified the antifibrotic action of XHC at the protein level (Fig. 8A to D). XHC significantly reduced the protein expression levels of the fibrosis markers α-SMA and collagen I in liver tissues. Concurrently, we confirmed at the cellular level the critical involvement of the PI3K/Akt signaling pathway in mediating XHC’s regulation of HSC activation, proliferation, apoptosis, and ECM synthesis. We employed the rat HSC line HSC-T6, a well-established model for HF research, for functional validation. Following 24 h of treatment with Xihuangcao (XHC), HSC-T6 cell viability remained unaffected at concentrations below 300 μg/ml, whereas 450 μg/ml significantly reduced viability (Fig. 8E). Consequently, concentrations of 125, 250, and 500 μg/ml were selected for subsequent experiments. Cell proliferation serves as a key indicator of HSC activation. Colony formation assays demonstrated reduced HSC proliferation, with a notable effect observed at 500 μg/ml (*P* < 0.05) (Fig. 8F). Flow cytometry revealed an increased apoptosis rate of HSC-T6 cells in response to escalating XHC concentrations, indicating that XHC promotes apoptosis in these cells (Fig. 8G). Compared with the control group, both migration (Fig. 8H) and invasion (Fig. 8I) of HSC-T6 cells were significantly suppressed following XHC treatment. These results collectively indicate that XHC inhibits HSC-T6 cell migration. Moreover, XHC dose-dependently reduced the mRNA and protein expression levels of the fibrosis markers α-SMA and collagen I in HSC-T6 cells (Fig. 8J), suggesting effective inhibition of HSC activation and ECM production. Based on KEGG pathway analysis, changes in mRNA and protein expression of PI3K/Akt signaling-related factors were verified using qRT-PCR and Western blotting. XHC significantly decreased PI3K and Akt gene expression in a dose-dependent manner. High- and medium-dose XHC treatment notably reduced the p-Akt/Akt protein ratio, whereas the low-dose group had no effect on this ratio (Fig. 8K), indicating that XHC may alleviate HF through inhibition of the PI3K/Akt signaling pathway. Additionally, XHC reduced PI3K/Akt protein expression levels in liver tissue (Fig. S4). Collectively, these findings suggest that XHC exerts antifibrotic effects via multitarget interactions, with prominent involvement of the PI3K/Akt pathway and suppression of HSC activation, proliferation, and invasion.

**Fig. 8.**
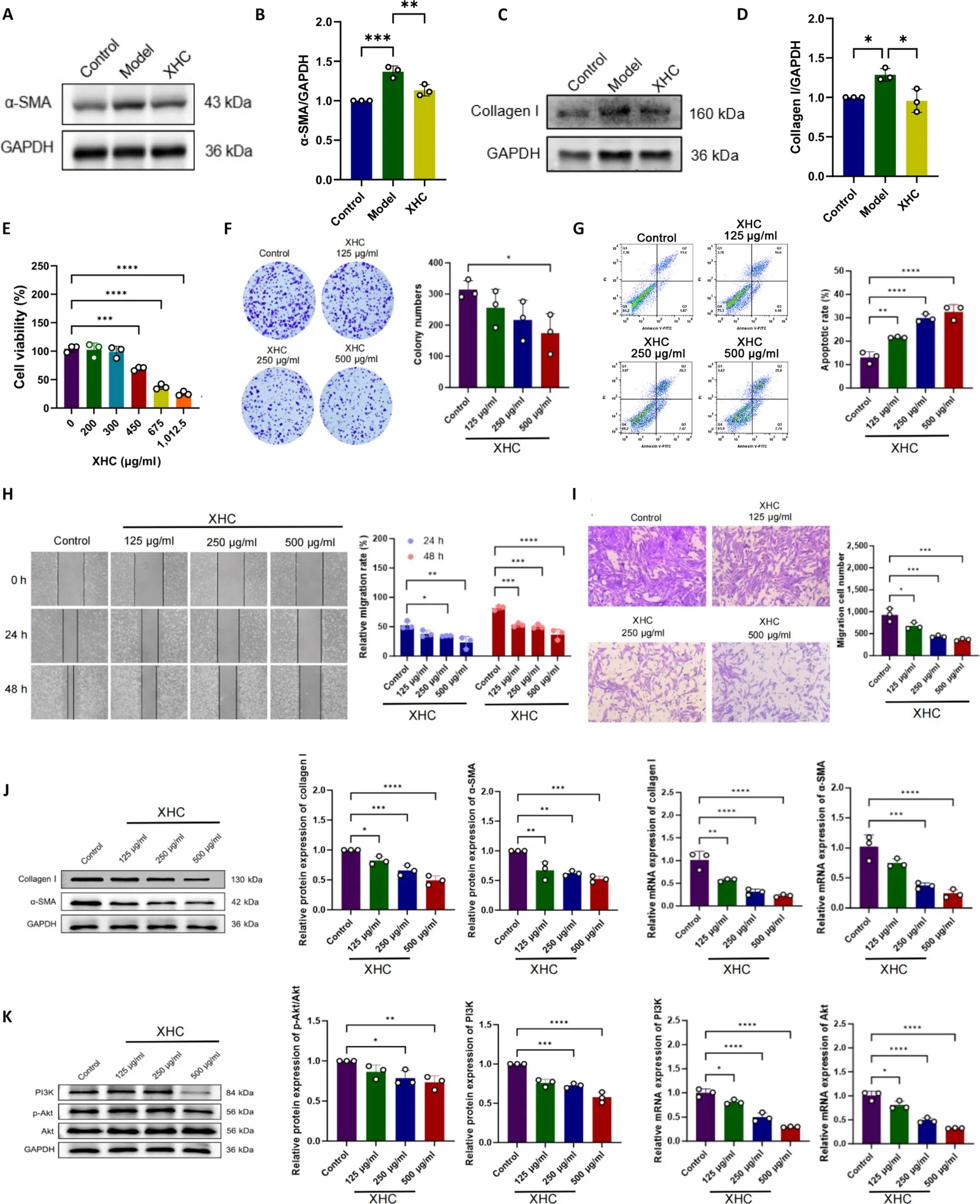
*Isodon serra* Kudô (XHC) mitigates hepatic fibrosis by inhibiting the PI3K/Akt signaling pathway. (A and B) Effect of XHC on the expression of alpha-smooth muscle actin (α-SMA) in liver tissue (*N* = 3). (C and D) Effect of XHC on the expression of collagen I in liver tissue (*N* = 3). (E) Effect of XHC on the viability of HSC-T6 cells (*N* = 3). (F) Image of HSC-T6 cell colony formation and statistical analysis of the number of clones in HSC-T6 cells (*N* = 3). (G) Flow cytometry image of HSC-T6 cell apoptosis and statistical analysis of the apoptosis rate of HSC-T6 cells (*N* = 3). (H) Scratch images of HSC-T6 cells and statistical analysis of the relative mobility of HSC-T6 cells (*N* = 3). (I) Invasion images of HSC-T6 cells and statistical analysis of the number of invasion of HSC-T6 cells (*N* = 3). (J) Effect of XHC on the expression of α-SMA and collagen I in HSC-T6 cells (*N* = 3). (K) Effect of XHC on the PI3K/Akt signaling pathway in HSC-T6 cells (*N* = 3). All data are shown as mean ± standard error of the mean (SEM).

## Discussion

Xihuangcao possesses diverse biological activities, with modern pharmacological studies confirming its ability to ameliorate liver disease in both animal and cellular models. In this work, a mouse model of carbon tetrachloride-induced HF was established to assess the antifibrotic effects of Xihuangcao using histological and multi-omics approaches. Our results showed that all 3 sources of Xihuangcao significantly attenuated HF. This was achieved through reduced inflammation and collagen deposition, down-regulation of profibrotic factors (TGF-β, PDGF, and TLR5), and modulation of several signaling pathways, including steroid biosynthesis, bile secretion, retinol metabolism, and the MAPK cascade. Metabolomic analysis indicated restoration of energy metabolism, amino acid turnover, and redox balance. Xihuangcao also ameliorated gut microbiota dysbiosis, notably by decreasing the abundance of Verrucomicrobia while increasing that of S24-7. Network pharmacology combined with experimental validation identified the PI3K/Akt pathway as a key mediator through which Xihuangcao inhibits HSC activation, proliferation, and invasion. Taken together, these findings furnish multi-omics evidence supporting the therapeutic potential of Xihuangcao and provide novel insights into its regulation of the gut–liver axis during HF.

Transcriptomic profiling of liver specimens demonstrated that Xihuangcao suppresses the expression of fibrosis-associated genes, including α-SMA and TGF-β, while concurrently modulating several metabolic pathways, namely, glycerophospholipid metabolism, steroid biosynthesis, bile secretion, and retinol metabolism. These transcriptomic alterations correlated well with observed metabolomic shifts, notably the restoration of BCAAs such as leucine and isoleucine, as well as ketone bodies including 3-hydroxybutyrate, acetone, and acetoacetate. These metabolites are recognized for their hepatoprotective and anti-inflammatory properties [18,19], acting through signaling cascades such as mTOR and Hcar2–cAMP [20]. Collectively, this hierarchical regulatory axis comprising the gut microbiota, the metabolic network, and gene transcription provides a robust molecular foundation for understanding how Xihuangcao reverses the pathological progression of HF.

Liver metabolomic analysis demonstrated that Xihuangcao reversed the abnormally elevated levels of α- and β-glucose in fibrotic livers, suggesting a potential role in enhancing insulin sensitivity and mitigating glucose-driven fibrogenesis. Furthermore, treatment with Xihuangcao restored the metabolic equilibrium of ketone bodies (3-hydroxybutyric acid, acetone, and acetoacetic acid) as well as pyruvate. These metabolites act synergistically to exert antifibrotic effects by regulating hepatic energy metabolism and suppressing HSC activation. Previous studies have established an association between insulin resistance and nonalcoholic fatty liver disease, which leads to elevated serum insulin and glucose concentrations [21]. Increased glucose levels promote the expression of connective tissue growth factor and type I collagen in HSCs, thereby contributing to fibrogenesis associated with nonalcoholic steatohepatitis [22]. The normalization of glucose and pyruvate metabolism by Xihuangcao likely improves energy homeostasis and alleviates hepatocyte stress. BCAAs were markedly depleted in the model group, consistent with prior reports indicating that reduced BCAA levels promote HSC activation via the MAPK pathway. Xihuangcao administration restored hepatic BCAA concentrations, thereby inhibiting MAPK activity through mTORC1 modulation and PPARα activation. This regulatory axis termed “BCAA restoration coupled with MAPK inhibition” represents a key antifibrotic mechanism of Xihuangcao.

Taurine represents the most abundant free amino acid in most animal tissues. Previous studies have demonstrated that taurine prevents HF through modulation of the TLR4/NF-κB signaling pathway, as well as by enhancing antioxidant defense mechanisms and reducing IL-6 expression, thereby regulating HSC activation [23]. Glutamine is synthesized from glutamic acid and ammonia via catalysis by glutamine synthetase. This reaction constitutes the primary pathway for ammonia transport and storage, playing a critical role in maintaining nitrogen balance in the body [24]. In the present experiment, glutamate levels were significantly reduced in the model group. Under ammonia-containing conditions, glutamate is converted to glutamine by glutamine synthetase, and glutamine is essential for preserving nitrogen homeostasis. The antioxidant properties of glutamine, along with its capacity to suppress α-SMA and TGF-β expression in liver tissue, have been reported to exert antifibrotic effects [25,26]. Clinical studies have revealed significantly reduced levels of both glutamine and glutamate in patients with HF [27]. In our experiment, serum ALT and AST concentrations were markedly elevated in the model group compared with those in the normal group. This elevation in aminotransferases is associated with hepatocellular injury, which leads to the release of glutamine into the bloodstream. Consequently, glutamine levels were significantly lower in the model group. The precise mechanism by which glutamine acts during organ damage remains incompletely understood. The protective effect of glutamine may be partially attributed to the preservation of glutathione content.

Recent advances in next-generation sequencing technology have revealed a strong link between gut dysbiosis and HF, providing new perspectives and potential intervention strategies for understanding and treating HF [28]. Consistent with this, we found significant alterations in gut microbial composition in CCl_4_-induced HF mice, particularly involving imbalances in Bacteroidetes, S24-7, and Verrucomicrobia [29,30]. Our study suggests that Xihuangcao supports liver health by promoting a microbiota structure associated with reduced inflammation and fibrosis. Meanwhile, Xihuangcao also decreased Verrucomicrobia abundance [31,32] and restored gut dysbiosis induced by HF.

The remodeling of gut microbiota and concomitant alterations in fecal metabolomics exhibited a synergistic effect. In the model group, the fecal levels of pro-inflammatory metabolites such as androstane-3α and 17β-diol were markedly elevated. Conversely, Xihuangcao suppressed the production of these compounds by modulating the microbial community structure while promoting the accumulation of antifibrotic metabolites, including the BCAAs leucine and isoleucine. BCAAs, comprising leucine, isoleucine, and valine, play important roles in liver diseases [33]. Previous studies have demonstrated that BCAAs effectively reverse CCl_4_-induced HF, improve glucose metabolism, and alleviate insulin resistance [34]. In the present study, leucine, isoleucine, and valine levels were significantly lower in the model group than in the control group. Administration of Xihuangcao significantly restored all 3 BCAA levels, suggesting that BCAAs may be associated with the regression of CCl_4_-induced HF in mice.

Spearman correlation analysis between 16S RNA-seq and metabolomics data screened by fecal and liver metabolomics revealed close connection between the gut microbial shifts and key hepatic/fecal differential metabolites [35,36]. Notably, *Blautia* and *Prevotella* correlated positively with BCAAs, suggesting their involvement in BCAA biosynthesis or conservation. Conversely, profibrotic metabolites such as androstan-3α,17β-diol were down-regulated following *Hedyotis* treatment, possibly due to remodeling of the structure of the microbiota. This highlights a synergistic “microbiota–metabolite–liver axis” as a central therapeutic route through which Xihuangcao exerts its protective effects against HF.

From a network pharmacology perspective, Xihuangcao’s antifibrotic effects arise from its multicomponent, multitarget regulatory actions. Key active compounds—flavonoids and diterpenoids—modulate critical signaling pathways such as PI3K/Akt, MAPK, and NF-κB. In vitro studies confirmed that Xihuangcao inhibits HSC activation by promoting Akt phosphorylation via PTEN suppression, leading to reduced expression of fibrosis markers like α-SMA and collagen I. This highlights PI3K/Akt as a central target in Xihuangcao’s mechanism of action.

In this study, HF models were established to evaluate the antifibrotic effects of the 3 botanical sources of Xihuangcao through histopathology, transcriptomics, metabolomics, 16S rRNA sequencing, and network pharmacology. In summary, this study presents integrated multi-omics evidence supporting the antifibrotic efficacy of Xihuangcao in a HF model. Xihuangcao alleviated liver fibrosis by suppressing inflammation and collagen deposition, down-regulating profibrotic factors (TGF-β, PDGF, and TLR5), and modulating pathways related to steroid biosynthesis, bile secretion, and MAPK signaling. It also restored energy metabolism, amino acid turnover, and redox balance at the metabolomic level while correcting gut dysbiosis—particularly by regulating Verrucomicrobia and S24-7. Network and experimental pharmacology further identified the PI3K/Akt pathway as a key target mediating its inhibition of HSC activation. These findings reveal the multitargeted and gut–liver axis-based mechanisms of Xihuangcao, providing not only modern scientific evidence for its traditional medicinal efficacy but also novel insights for developing therapeutic strategies targeting the “gut–liver axis” in HF.

Although this study confirmed that the 3 different sources of Xihuangcao all exhibited significant anti-liver fibrosis effects as a whole, their respective unique chemical composition characteristics may potentially lead to subtle differences in the intensity and mechanism of action of the efficacy. Therefore, in the future, it is necessary to further carry out systematic chemical component comparisons and corresponding pharmacological comparative studies to more precisely clarify the specific contributions of different sources of Xihuangcao in the antifibrotic effect and its potential differentiated mechanisms.

It is important to note that the CCl_4_ model used here has inherent limitations, as it models a specific, toxin-induced form of fibrosis. To comprehensively validate the therapeutic potential of Xihuangcao, future studies would benefit from employing complementary models, such as bile duct ligation for cholestatic injury or diet-induced nonalcoholic steatohepatitis models, which more closely mimic the metabolic and progressive nature of human fibrogenesis.

## Data Availability

The data are available from the corresponding authors on reasonable request.
