# Supplementary material for: Multi-omics Profiling Reveals the Effects of Xihuangcao on Hepatic Fibrosis Attenuation through Gut Microbiota and Metabolic Reprogramming
Source: Biomater Res. 2026 Oct 19;30:0422. doi: 10.34133/bmr.0422 (PMC13678083; doi:10.34133/bmr.0422)
Supplement: Supplementary file 1 [file bmr.0422.f1.docx]

**Supporting information**

**Multi-Omics Profiling Reveals the Effects of Xihuangcao on Hepatic Fibrosis Attenuation through Gut Microbiota and Metabolic Reprogramming**

Weiguo Zhao^1 #^, Xueling Zhang^2 #^, Lifan Xu^2 #^, Mianfeng Zheng^3 #^, Zhuoya Li^1^, Yuexuan Wang^2^, Xinxiang He^4^, Min Hong^5^, Huiye Zhang^6^, Junwei Li^6^, Lei Shi^2^, Ying Du^7^, Haitao Yuan^2 *^, Wei Zhang^2 *^, Ali Chen^2 *^

^1^ Department of Pharmacy, Zhongshan Municipal People’s Hospital, Zhongshan, 528403

^2^ Center for Drug Research and Development, Guangdong Provincial Key Laboratory of Advanced Drug Delivery System, Guangdong Pharmaceutical University, Guangzhou, 510006

^3^ Key Laboratory of Glucolipid Metabolic Disorder, Ministry of Education, Guangdong Pharmaceutical University, Guangzhou 510006, China.

^4^ Department of Gastroenterology, The First Affiliated Hospital of Guangdong Pharmaceutical University, Guangzhou 510080, Guangdong Province, China

^5^ The First Affiliated Hospital of Guangdong Pharmaceutical University, Guangzhou, 510080

^6^ Hutchison Whampoa Guangzhou Baiyunshan, Chinese Medicine Company Limited, Guangzhou, 510006

^7^ Consun Pharmaceutical Group Limited, Guangzhou, Guangdong, 510006

^#^ These authors contributed equally to this work.

*Corresponding authors: Ali Chen (chenali@gdpu.edu.cn); Wei Zhang (zhangweia@gdpu.edu.cn); Haitao Yuan (yht193525@163.com)

**Materials and methods**

**Biochemical measurements in serum**

Measure the serum levels of alanine aminotransferase (ALT) and aspartate aminotransferase (AST) were assayed using commercially available kits (Nanjing jiancheng, China), adhering strictly to the manufacturer's guidelines for assay performance.

**Histological analysis**

Fresh livers were fixed in 4% paraformaldehyde for 36-48 hours. The tissues underwent dehydration, embedding in paraffin and sectioned into thin slices of 5 µm. These sections were stained, including hematoxylin and eosin (H&E) stain, Masson's trichrome stain, Sirius Red stain, and immunohistochemistry (IHC).

**Reverse Transcription-Polymerase Chain Reaction (RT-PCR) assay**

Total RNA was extracted using TRIzol reagent (Vazyme, Nanjing, China) and reverse transcribed using a reverse transcription kit (Vazyme, Nanjing, China). cDNA amplification was performed on a Light Cycler 96 (Roche) using specific primers and SYBR Green PCR premix (Toyobo, Osaka, Japan). The relative mRNA expression levels were quantitatively determined by the Comparative Ct method, with normalization performed using the amount of 18S rRNA in each sample as a reference.

**Metabolomics experiments**

Approximately 200 mg of liver tissue was thawed on ice with 300 μL ultrapure water and 600 μL methanol. After sonication in an ice-water bath, the mixture was centrifuged (13,000 rpm, 10 min, 4℃). The supernatant was nitrogen-dried. For ^1^H-NMR analysis, 150 μL PBS (0.2 mol/L, pH 7.4) and 400 μL TSP-containing D_2_O were added, vortexed, and centrifuged again (13,000 rpm, 10 min, 4℃). The final supernatant was transferred to a 5-mm NMR tube.

Fecal samples (50 mg) were extracted with 1 mL of methanol:acetonitrile:water (2:2:1, containing 20 mg/L internal standard), vortexed (30 s), ground (45 Hz, 10 min) with steel balls, and ultrasonicated (ice-water bath, 10 min). After standing at -20℃ for 1 h, the mixture was centrifuged (12,000 rpm, 15 min, 4℃). The supernatant was vacuum-dried, redissolved in acetonitrile:water (1:1), vortexed (30 s), ultrasonicated (10 min, ice-water bath), and centrifuged again (12,000 rpm, 15 min, 4℃). The final supernatant was collected for analysis.

Sample size (n=6 per group) was determined based on power analysis (α = 0.05, power = 0.8) using preliminary data on serum ALT levels, and is consistent with widely accepted standards for multi-omics studies in murine fibrosis models. The same six mice per group were used for all omics analyses to maintain consistency, except when samples were lost due to technical issues (as indicated in figure legends).

**Fig. S1**. The toxicological experiment in mice of XHC and Colchicine. (A-B) Serum ALT levels in mice (N = 5-8). (C-D) Serum AST levels in mice (N = 5-8). (E) HE and Masson staining diagram of liver tissue (N = 5-8). (F)Statistical chart of Masson staining of liver tissue (N = 6-8). All data are shown as the mean ± standard error of the mean. Scale bars, 100 μm.

**Fig. S2.** (A) Xihuangcao restores liver metabolites of mice with hepatic fibrosis and multivariate analysis of liver metabolomics. Abbreviations XW, XH, and XHC are defined in Fig. 1. Representative ^1^H-NMR spectra of the liver of three origins of Xihuangcao.

**Fig. S3**. (A) XHC restores fecal metabolites of mice with hepatic fibrosis. TIC chromatogram of each group in fecal.

**Fig. S4.** Effect of XHC on PI3K/Akt signaling pathway in liver tissue. (A)Western blotting bands of PI3K, p-Akt, and the internal control GAPDH in each group. (B)Statistical analysis of the relative expression level of PI3K protein (N = 3). (C)Statistical analysis of the relative expression level of p-Akt protein (N = 3). All data are shown as the mean ± standard error of the mean.

**Table**

Table S1 Antibody information

| **Antibodies** | **Sources** | **Identifier** | **Dilution ratios** |
| --- | --- | --- | --- |
| α-SMA antibody | proteintech | Cat#14395-1-AP | 1:6000 |
| Collagen I antibody | proteintech | Cat#66761-1-Ig | 1:5000 |
| PI3K antibody | proteintech | Cat#82796-4-RR | 1:10000 |
| Akt antibody | proteintech | Cat# 10176-2-AP | 1:1000 |
| p-Akt antibody | proteintech | Cat# 66444-1-Ig | 1:5000 |
| GAPDH polyclonal antibody | proteintech | Cat# 10494-1-AP | 1:4000 |
| Anti-rabbit IgG, HRP-linked antibody | Cell Signaling Technology | Cat# 7074 | 1:10000 |

Table S2 Potential biomarker information for liver tissue

| No | Metabolites | Model/Ctrl | | | | XHC/Model | | | |
| --- | --- | --- | --- | --- | --- | --- | --- | --- | --- |
|  |  | VIP | FC | P | trend | VIP | FC | P | trend |
| 1 | α-Glucose | 5.54 | 1.63 | ** | ↑ | 10.01 | 1.80 | 0.11 | ↓ |
| 2 | β-Glucose | 1.06 | 0.92 | *** | ↑ | 4.53 | 1.35 | * | ↓ |
| 3 | Taurine | 2.27 | 0.84 | ** | ↓ | 1.68 | 0.90 | 0.81 | ↑ |
| 4 | Leucine | 2.40 | 1.28 | * | ↑ | 3.71 | 0.71 | *** | ↑ |
| 5 | Glutamate | 3.81 | 0.74 | 0.06 | ↓ | 0.21 | 0.87 | 0.11 | ↑ |
| 6 | Isoleucine | 2.11 | 0.76 | ** | ↓ | 1.35 | 0.87 | 0.78 | ↓ |
| 7 | Alanine | 1.64 | 1.16 | ** | ↓ | 2.90 | 0.87 | 0.78 | ↑ |
| 8 | Valine | 2.11 | 0.70 | ** | ↓ | 1.91 | 0.72 | 0.68 | ↑ |
| 9 | Lysine | 2.39 | 1.94 | *** | ↓ | 2.89 | 0.55 | 0.92 | ↓ |
| 10 | Glycine | 1.09 | 0.66 | ** | ↓ | 1.99 | 1.60 | 0.96 | ↑ |
| 11 | GPC | 1.53 | 0.74 | * | ↑ | 1.73 | 1.30 | 0.25 | ↑ |
| 12 | GSSG | 1.01 | 0.85 | * | ↓ | 1.11 | 1.26 | 0.64 | ↓ |
| 13 | Dimethylglycine | 3.62 | 0.50 | ** | ↑ | 0.61 | 0.96 | 0.66 | ↓ |
| 14 | 3-Hydroxybutyrate | 1.19 | 0.70 | * | ↓ | 2.56 | 0.73 | 0.32 | ↑ |
| 15 | Acetoacetate | 2.26 | 1.66 | * | ↓ | 2.82 | 0.59 | 0.78 | ↓ |
| 16 | Butyrate | 1.34 | 1.27 | * | ↓ | 7.29 | 0.58 | 0.18 | ↓ |
| 17 | Pyruvate | 1.27 | 0.79 | **** | ↓ | 1.34 | 0.78 | 0.92 | ↓ |
| 18 | Acetone | 1.25 | 0.46 | *** | ↓ | 2.03 | 1.49 | 0.98 | ↓ |
| 19 | Creatine | 1.15 | 0.57 | ** | ↓ | 1.02 | 0.67 | * | ↑ |
| 20 | Succinate | 1.63 | 0.71 | ** | ↓ | 1.34 | 0.78 | 0.80 | ↑ |
| 21 | Formate | 1.07 | 1.72 | * | ↓ | 1.15 | 0.63 | * | ↑ |

Table S3 Metabolic pathway analysis of liver tissue

| key | Pathway Name | Match Status | -log(p) | Impact |
| --- | --- | --- | --- | --- |
| 1 | Butanoate metabolism | [4/15](https://dev.metaboanalyst.ca/Secure/pathway/PathResultView.xhtml) | 4.787 | 0.111 |
| 2 | Valine, leucine and isoleucine biosynthesis | [3/8](https://dev.metaboanalyst.ca/Secure/pathway/PathResultView.xhtml) | 4.143 | 0.000 |
| 3 | Alanine, aspartate and glutamate metabolism | [4/28](https://dev.metaboanalyst.ca/Secure/pathway/PathResultView.xhtml) | 3.652 | 0.197 |
| 4 | Glyoxylate and dicarboxylate metabolism | [4/32](https://dev.metaboanalyst.ca/Secure/pathway/PathResultView.xhtml) | 3.420 | 0.083 |
| 5 | Glycine, serine and threonine metabolism | [4/34](https://dev.metaboanalyst.ca/Secure/pathway/PathResultView.xhtml) | 3.316 | 0.354 |
| 6 | Valine, leucine and isoleucine degradation | [4/40](https://dev.metaboanalyst.ca/Secure/pathway/PathResultView.xhtml) | 3.041 | 0.000 |
| 7 | Glutathione metabolism | [3/28](https://dev.metaboanalyst.ca/Secure/pathway/PathResultView.xhtml) | 2.440 | 0.135 |
| 8 | Arginine and proline metabolism | [3/36](https://dev.metaboanalyst.ca/Secure/pathway/PathResultView.xhtml) | 2.127 | 0.024 |
| 9 | Citrate cycle (TCA cycle) | [2/20](https://dev.metaboanalyst.ca/Secure/pathway/PathResultView.xhtml) | 1.667 | 0.079 |
| 10 | Neomycin, kanamycin and gentamicin biosynthesis | [1/2](https://dev.metaboanalyst.ca/Secure/pathway/PathResultView.xhtml) | 1.635 | 0.000 |
| 11 | One carbon pool by folate | [2/26](https://dev.metaboanalyst.ca/Secure/pathway/PathResultView.xhtml) | 1.451 | 0.113 |
| 12 | Lipoic acid metabolism | [2/28](https://dev.metaboanalyst.ca/Secure/pathway/PathResultView.xhtml) | 1.392 | 0.002 |
| 13 | Porphyrin metabolism | [2/31](https://dev.metaboanalyst.ca/Secure/pathway/PathResultView.xhtml) | 1.311 | 0.000 |

Table S4 Information on potential biomarkers in fecal (positive ion mode)

| POS | | | | | | | | | | |  |
| --- | --- | --- | --- | --- | --- | --- | --- | --- | --- | --- | --- |
| No | Metabolites | Ctrl_vs_Model | | | |  | Model_vs_XHC | | | |  |
|  |  | log2FoldChange | P | VIP | Trend |  | log2FoldChange | P | VIP | Trend | |
| 1 | Cholestanetriol | 0.86 | ** | 1.81 | ↑ |  | -1.55 | *** | 2.38 | ↓ | |
| 2 | PE(16:1(9Z)/16:1(9Z)) | 0.44 | ** | 1.71 | ↑ |  | -0.34 | * | 1.70 | ↓ | |
| 3 | Polymyxin M | -0.72 | * | 1.50 | ↓ |  | -0.63 | * | 2.06 | ↓ | |
| 4 | alpha-[3-(Nitrosoamino)propyl]-3-pyridinemethanol | 1.31 | * | 1.74 | ↑ |  | -0.41 | * | 1.72 | ↓ | |
| 5 | Antibiotic G-418 | 0.67 | * | 1.50 | ↑ |  | -0.44 | ** | 2.14 | ↓ | |
| 6 | Nalidixic Acid | 0.53 | * | 1.71 | ↑ |  | -0.24 | * | 2.01 | ↓ | |
| 7 | D,L- gamma-hydroxyphosphinothricin | 1.54 | ** | 1.85 | ↑ |  | -0.78 | * | 1.81 | ↓ | |
| 8 | Benzenepropanenitrile | -1.05 | ** | 1.87 | ↓ |  | 0.82 | * | 1.75 | ↑ | |
| 9 | (R)-3,7-Dimethyl-5-indanecarboxylic acid | -0.78 | * | 1.77 | ↓ |  | 0.29 | * | 1.93 | ↑ | |
| 10 | Ethyl vanillin | 0.47 | * | 1.51 | ↑ |  | -0.49 | * | 2.02 | ↓ | |
| 11 | DIMETHYLCAFFEIC ACID | 1.08 | * | 1.76 | ↑ |  | -0.93 | ** | 2.24 | ↓ | |
| 12 | Coniferyl alcohol | 0.70 | * | 1.65 | ↑ |  | -0.63 | *** | 2.33 | ↓ | |
| 13 | geneticin | 0.38 | ** | 1.79 | ↑ |  | -0.35 | * | 1.92 | ↓ | |
| 14 | 2,4,6-octatrienal | -3.13 | * | 1.74 | ↓ |  | 0.95 | * | 1.74 | ↑ | |
| 15 | (±)-Dulciol E | 0.63 | * | 1.54 | ↑ |  | -0.64 | *** | 2.40 | ↓ | |
| 16 | Dhurrin 6'-glucoside | 0.47 | * | 1.59 | ↑ |  | -0.38 | * | 1.88 | ↓ | |
| 17 | (1S,4R)-p-Mentha-2,8-dien-1-ol | 0.43 | ** | 1.84 | ↑ |  | -0.52 | * | 2.02 | ↓ | |
| 18 | LysoPC(14:0/0:0) | 0.52 | ** | 1.82 | ↑ |  | -0.37 | * | 1.92 | ↓ | |
| 19 | Lysyl-Asparagine | 2.14 | ** | 1.76 | ↑ |  | -1.38 | * | 1.70 | ↓ | |
| 20 | OA-6129 A | 0.43 | * | 1.49 | ↑ |  | -0.58 | * | 1.81 | ↓ | |
| 21 | 19-Chloroproansamitocin | 0.74 | * | 1.57 | ↑ |  | -0.56 | * | 1.68 | ↓ | |
| 22 | 4-sec-Butylphenol | -0.39 | ** | 1.85 | ↓ |  | 0.33 | * | 2.03 | ↑ | |
| 23 | Fumiquinazoline F-indoline-2',3'-diol | 2.68 | * | 1.76 | ↑ |  | -3.13 | * | 2.07 | ↓ | |
| 24 | Epothilone A | 0.46 | ** | 1.77 | ↑ |  | -0.60 | * | 1.98 | ↓ | |
| 25 | PE(P-18:0/18:3(9,11,15)-OH(13)) | 0.91 | * | 1.52 | ↑ |  | -0.83 | * | 1.75 | ↓ | |
| 26 | 17-Hydroxymethylethisterone | 1.96 | * | 1.86 | ↑ |  | -1.29 | * | 1.88 | ↓ | |
| 27 | 4-Fluorocyclohexane-1-carboxylic acid | 2.02 | ** | 1.98 | ↑ |  | -0.79 | * | 1.84 | ↓ | |
| 28 | PG(17:0/16:1(9Z)) | 2.03 | * | 1.67 | ↑ |  | -2.47 | * | 2.07 | ↓ | |
| 29 | 5,7-nonadienoic acid | -0.71 | * | 1.61 | ↓ |  | 0.67 | * | 1.80 | ↑ | |
| 30 | Undecylenic acid | 1.17 | * | 1.67 | ↑ |  | -1.37 | * | 2.03 | ↓ | |
| 31 | (±)-Myrtenyl acetate | 1.04 | * | 1.68 | ↑ |  | -1.00 | * | 1.90 | ↓ | |
| 32 | Glutinosone | 1.34 | * | 1.61 | ↑ |  | -1.59 | * | 1.92 | ↓ | |
| 33 | Androstan-3alpha,17beta-diol | 1.93 | * | 1.68 | ↑ |  | -1.80 | * | 1.83 | ↓ | |
| 34 | 8(S)-HEPE | 1.01 | * | 1.69 | ↑ |  | -0.83 | * | 1.75 | ↓ | |
| 35 | N-Oleoyl Glutamine | 2.48 | * | 1.52 | ↑ |  | -4.67 | * | 2.01 | ↓ | |
| 36 | 20-COOH-Leukotriene B4 | -0.26 | * | 1.70 | ↓ |  | 0.93 | * | 1.96 | ↑ | |
| 37 | 20-HETE ethanolamide | 1.38 | * | 1.88 | ↑ |  | -0.78 | * | 1.86 | ↓ | |
| 38 | DG(15:0/PGF1alpha/0:0) | 2.20 | * | 2.08 | ↑ |  | -1.03 | ** | 2.17 | ↓ | |
| 39 | 4-hydroxyestradiol | 2.37 | * | 1.67 | ↑ |  | -1.67 | * | 1.80 | ↓ | |
| 40 | 2,4,6-Trimethylbenzaldehyde | 0.71 | * | 1.69 | ↑ |  | -0.46 | * | 1.72 | ↓ | |
| 41 | DG(20:3(8Z,11Z,14Z)-2OH(5,6)/16:0/0:0) | 2.34 | * | 1.80 | ↑ |  | -1.58 | * | 1.92 | ↓ | |
| 42 | 17beta-Hydroxy-2alpha-methyl-5alpha-estran-3-one | -0.59 | ** | 1.78 | ↓ |  | 0.39 | * | 1.90 | ↑ | |
| 43 | Mevalonolactone | 0.29 | *** | 2.04 | ↑ |  | -0.14 | * | 1.94 | ↓ | |
| 44 | 1-Octen-3-yl glucoside | 0.13 | * | 1.50 | ↑ |  | -0.14 | ** | 2.15 | ↓ | |
| 45 | Tempo-maleimide | 0.57 | * | 1.70 | ↑ |  | -0.54 | * | 1.84 | ↓ | |

Table S5 Information on potential biomarkers in fecal (negative ion mode)

| NEG | | | | | | | | | | | | | |
| --- | --- | --- | --- | --- | --- | --- | --- | --- | --- | --- | --- | --- | --- |
| No | Metabolites | | Ctrl_vs_Model | | | | |  | Model_vs_XHC | | | | |
|  |  |  | log2FoldChange | P | VIP | Trend | |  | Fold Change | P | VIP | Trend | |
| 1 | | Methylphosphonate | 0.183 | ** | 1.811 | | ↑ |  | -0.151 | ** | 1.952 | | ↓ |
| 2 | | Ethosuximide | -0.410 | * | 1.581 | | ↓ |  | 0.382 | * | 1.808 | | ↑ |
| 3 | | Quetiapine | 1.655 | ** | 1.902 | | ↑ |  | -0.911 | * | 1.786 | | ↓ |
| 4 | | Creatine | -1.103 | * | 1.535 | | ↓ |  | 0.153 | * | 1.654 | | ↑ |
| 5 | | 6,7-Dimethyl-8-(1-D-ribityl)lumazine | 0.469 | * | 1.410 | | ↑ |  | -0.380 | * | 1.612 | | ↓ |
| 6 | | 2'-Fluoro-2',3'-dideoxyinosine | 0.855 | * | 1.633 | | ↑ |  | -1.055 | *** | 2.225 | | ↓ |
| 7 | | Zalcitabine | 0.700 | * | 1.395 | | ↑ |  | -0.648 | * | 1.623 | | ↓ |
| 8 | | Pirfenidone | -0.489 | * | 1.696 | | ↓ |  | 0.452 | * | 1.637 | | ↑ |
| 9 | | 9-(Dimethylallyl)-5,10-dihydrophenazine-1-carboxylate | 0.631 | * | 1.432 | | ↑ |  | -0.788 | ** | 2.194 | | ↓ |
| 10 | | Glutaminyl-Gamma-glutamate | 0.726 | * | 1.553 | | ↑ |  | -0.722 | * | 1.670 | | ↓ |
| 11 | | Irtemazole | -1.826 | ** | 1.892 | | ↓ |  | -0.423 | * | 1.657 | | ↓ |
| 12 | | 3-(O-Geranylgeranyl)-sn-glycerol 1-phosphate | 0.319 | * | 1.490 | | ↑ |  | 0.678 | * | 1.863 | | ↑ |
| 13 | | Desglucocoroloside | 0.699 | ** | 1.881 | | ↑ |  | 0.454 | * | 1.759 | | ↑ |
| 14 | | 2-n-heptyl-4-hydroxyquinoline-N-oxide | -0.824 | * | 1.583 | | ↓ |  | 0.395 | * | 1.675 | | ↑ |
| 15 | | (2E,11Z)-Wyerone acid | -0.527 | ** | 1.748 | | ↓ |  | -0.609 | * | 1.706 | | ↓ |
| 16 | | Epirosmanol | -1.856 | * | 1.768 | | ↓ |  | 6.171 | * | 2.369 | | ↑ |
| 17 | | Tabersonine | -1.924 | * | 1.495 | | ↓ |  | 1.310 | * | 1.876 | | ↑ |
| 18 | | Dehydrocurdione | 1.278 | * | 1.421 | | ↑ |  | -1.598 | * | 1.814 | | ↓ |
| 19 | | 11,12-DHET | -0.858 | * | 1.391 | | ↓ |  | 0.823 | * | 1.657 | | ↑ |
| 20 | | Panaquinquecol 1 | -0.829 | *** | 1.956 | | ↓ |  | -0.611 | * | 1.636 | | ↓ |
| 21 | | 5'-Carboxy-gamma-chromanol | -2.390 | * | 1.522 | | ↓ |  | 1.074 | * | 1.762 | | ↑ |
| 22 | | Desoxycorticosterone pivalate | 0.806 | * | 1.500 | | ↑ |  | -1.388 | ** | 2.024 | | ↓ |
| 23 | | PG(19:0/19:0) | 0.989 | * | 1.505 | | ↑ |  | -1.547 | * | 2.035 | | ↓ |
| 24 | | 9-Hydroxy-12-oxo-10(E),15(Z)-octadecadienoic acid | -1.087 | * | 1.546 | | ↓ |  | 3.658 | ** | 2.167 | | ↑ |
| 25 | | (10S)-Juvenile hormone III diol | -0.817 | * | 1.876 | | ↓ |  | -1.108 | ** | 1.951 | | ↓ |
| 26 | | 1-palmitoyl-dihydroxyacetone-phosphate | -0.619 | * | 1.465 | | ↓ |  | 0.895 | * | 1.720 | | ↑ |
| 27 | | (±)p-CMBHC | -0.799 | * | 1.566 | | ↓ |  | -1.226 | * | 1.742 | | ↓ |
| 28 | | (S)-Carvone | -0.866 | *** | 2.019 | | ↓ |  | 0.778 | *** | 2.389 | | ↑ |
| 29 | | 11-Deoxycortisol | -0.620 | ** | 1.877 | | ↓ |  | 0.587 | ** | 2.133 | | ↑ |
| 30 | | D-Fructose | 0.666 | ** | 1.715 | | ↑ |  | -0.631 | ** | 1.893 | | ↓ |
| 31 | | Galactonic aci | 0.789 | * | 1.675 | | ↑ |  | -0.725 | ** | 2.058 | | ↓ |
| 32 | | 2-octaprenylphenol | 1.209 | * | 1.583 | | ↑ |  | -0.870 | * | 1.661 | | ↓ |
| 33 | | 3,4-Dihydroanhydrorhodovibrin | 0.385 | * | 1.454 | | ↑ |  | 0.329 | * | 1.637 | | ↑ |
| 34 | | (S)-2,3,4,5-Tetrahydropyridine-2-carboxylate | -0.906 | * | 1.522 | | ↓ |  | 0.321 | * | 1.739 | | ↑ |
| 35 | | 3-Hydroxynorvaline | 1.339 | ** | 1.913 | | ↑ |  | -0.617 | ** | 2.036 | | ↓ |
| 36 | | Isopenicillin N | 0.650 | * | 1.910 | | ↑ |  | -0.513 | * | 1.920 | | ↓ |
| 37 | | N,N-Dimethyl-5-({2-Methyl-6-[(5-Methylpyrazin-2-Yl)carbamoyl]-1-Benzofuran-4-Yl}oxy)pyrimidine-2-Carboxamide | 1.111 | *** | 2.020 | | ↑ |  | -1.072 | ** | 2.054 | | ↓ |

Table S6 Metabolic pathway analysis of fecal

| key | Pathway Name | Match Status | -log(p) | Impact |
| --- | --- | --- | --- | --- |
| 1 | Glycerophospholipid metabolism | [2/36](https://new.metaboanalyst.ca/Secure/pathway/PathResultView.xhtml) | 1.8679* | 0.122 |
| 2 | Steroid hormone biosynthesis | [2/79](https://new.metaboanalyst.ca/Secure/pathway/PathResultView.xhtml) | 1.2275 | 0.016 |
| 3 | Starch and sucrose metabolism | 1/15 | 1.1232 | 0.007 |
| 4 | Fructose and mannose metabolism | 1/18 | 1.0470 | 0.098 |
| 5 | Galactose metabolism | 1/27 | 0.8797 | 0.000 |
| 6 | Lysine degradation | 1/30 | 0.8369 | 0.000 |
| 7 | Glycosylphosphatidylinositol (GPI)-anchor biosynthesis | [1/32](https://new.metaboanalyst.ca/Secure/pathway/PathResultView.xhtml) | 0.8108 | 0.037 |
| 8 | Glycine, serine and threonine metabolism | [1/34](https://new.metaboanalyst.ca/Secure/pathway/PathResultView.xhtml) | 0.7865 | 0.000 |
| 9 | Arginine and proline metabolism | [1/36](https://new.metaboanalyst.ca/Secure/pathway/PathResultView.xhtml) | 0.7636 | 0.024 |
| 10 | Amino sugar and nucleotide sugar metabolism | [1/42](https://new.metaboanalyst.ca/Secure/pathway/PathResultView.xhtml) | 0.7025 | 0.000 |
| 11 | Arachidonic acid metabolism | [1/43](https://new.metaboanalyst.ca/Secure/pathway/PathResultView.xhtml) | 0.6932 | 0.000 |

Table S7 Spearman correlation analysis of metabolites and intestinal microbiota

|  | Blautia | Paraprevotella | Coprococcus | Prevotella | Desulfovibrio | Clostridium |
| --- | --- | --- | --- | --- | --- | --- |
| PE(16:1(9Z)/16:1(9Z)) | 0.726** | 0.835** | 0.813** | -0.314 | 0.228 | 0.649* |
| LysoPC(14:0/0:0) | -0.645* | -0.669* | -0.734* | 0.115 | 0 | -0.571 |
| Androstan-3alpha,17beta-diol | -0.833** | -0.669* | -0.709* | 0.479 | -0.087 | -0.517 |
| Creatine | 0.768** | 0.448 | 0.59 | -0.406 | -0.087 | 0.328 |
| 1112-DHET | 0.495 | 0.423 | 0.452 | -0.37 | 0.609 | 0.383 |
| 11-Deoxycortisol | 0.768** | 0.791** | 0.703* | -0.6 | 0.696* | 0.644* |
| D-Fructose | -0.866** | -0.718* | -0.684* | 0.648* | -0.522 | -0.650* |
| (S)-2345-Tetrahydropyridine-2-carboxylate | 0.794** | 0.472 | 0.759* | -0.236 | 0.087 | 0.426 |
| α-Glucose | -0.408 | -0.513 | -0.287 | 0.806** | -0.437 | -0.526 |
| β-Glucose | -0.54 | -0.644* | -0.447 | 0.855** | -0.488 | -0.646* |
| Leucine | 0.116 | 0.281 | -0.065 | -0.539 | 0.198 | 0.361 |
| Glutamate | 0.348 | 0.406 | 0.189 | -0.661* | 0.644* | 0.498 |
| Isoleucine | -0.266 | -0.071 | -0.465 | -0.491 | -0.032 | -0.021 |
| Alanine | 0.375 | 0.452 | 0.262 | -0.697* | 0.147 | 0.474 |
| Valine | 0.232 | 0.424 | 0.113 | -0.636* | 0.133 | 0.453 |
| Glycine | 0.022 | 0.206 | -0.084 | -0.770** | 0.055 | 0.298 |
| GSSG | -0.082 | -0.164 | -0.327 | -0.418 | 0.226 | 0.053 |
| Dimethylglycine | -0.12 | -0.317 | 0.04 | 0.758* | -0.258 | -0.326 |
| Acetoacetate | -0.393 | -0.185 | -0.505 | -0.309 | 0.308 | -0.119 |
| Butyrate | -0.528 | -0.534 | -0.548 | -0.042 | -0.032 | -0.214 |
| Pyruvate | -0.225 | -0.05 | -0.28 | -0.212 | 0.488 | 0.06 |
| Creatine | 0.637* | 0.537 | 0.414 | -0.758* | 0.377 | 0.568 |
| Succinate | 0.513 | 0.602* | 0.352 | -0.903** | 0.552 | 0.596* |
| Succinate | 0.671* | 0.680* | 0.548 | -0.309 | 0.212 | 0.702* |

Table S8 Spearman correlation analysis between fecal metabolites and liver metabolites

|  | PE(16:1(9Z)/16:1(9Z)) | LysoPC(14:0/0:0) | Androstan-3alpha,17beta-diol | Creatine | 1112-DHET | 11-Deoxycortisol | D-Fructose | (S)-2345-Tetrahydropyridine-2-carboxylate |
| --- | --- | --- | --- | --- | --- | --- | --- | --- |
| α-Glucose | 0.285 | -0.103 | 0.491 | -0.248 | -0.067 | -0.612 | 0.030 | -0.188 |
| β-Glucose | 0.236 | 0.030 | 0.515 | -0.273 | -0.236 | -0.697 | 0.152* | -0.236 |
| Leucine | -0.636 | 0.236 | -0.261 | 0.042 | -0.248 | 0.273 | 0.079 | -0.103 |
| Glutamate | -0.200 | 0.018 | -0.515 | 0.212 | -0.309 | 0.406 | 0.200 | 0.152 |
| Isoleucine | -0.394 | 0.685* | 0.067 | -0.115 | -0.212 | -0.067 | 0.212 | -0.406 |
| Alanine | -0.576 | 0.236 | -0.382 | 0.152 | 0.067 | 0.588 | -0.188 | 0.176 |
| Valine | -0.503 | 0.455 | -0.115 | -0.042 | -0.055 | 0.297 | 0.055 | -0.164 |
| Glycine | -0.261 | 0.564 | -0.091 | -0.006 | 0.042 | 0.261 | -0.030 | 0.018 |
| GSSG | -0.164 | 0.600 | 0.164 | -0.212 | -0.115 | -0.103 | 0.333 | -0.503 |
| Dimethylglycine | 0.164 | -0.333 | 0.309 | -0.067 | 0.139 | -0.370 | -0.176 | 0.006 |
| Acetoacetate | 0.370 | 0.406 | 0.103 | -0.176 | -0.515 | -0.345 | 0.685 | -0.394* |
| Butyrate | 0.152 | 0.418 | 0.176 | -0.236 | -0.576 | -0.370 | 0.552 | -0.200 |
| Pyruvate | 0.442 | 0.103 | -0.127 | 0.006 | -0.539 | -0.248 | 0.515 | -0.067 |
| Creatine | -0.612 | -0.164 | -0.515 | 0.297 | 0.321 | 0.770* | -0.309 | 0.224 |
| Succinate | -0.103 | 0.212 | -0.321 | 0.115 | 0.285 | 0.576 | -0.055 | 0.018 |
| Formate | -0.055 | -0.188 | 0.115 | -0.418 | 0.224 | 0.370 | -0.018 | -0.200 |
